# Supplementary material for: Partitioning variability in animal behavioral videos using semi-supervised variational autoencoders
Source: PLoS Comput Biol. 2021 Sep 22;17(9):e1009439. doi: 10.1371/journal.pcbi.1009439 (PMC8489729; doi:10.1371/journal.pcbi.1009439)
Supplement: S1 Fig — A: Top: The red box outlines the frame crop used to compute a hand engineered whisker pad feature. We perform PCA on this cropped portion of each frame and take the first principal component (PC) as a proxy for whisker pad position. Bottom: The PS-VAE unsupervised latent corresponding to the whisker pad (Fig 4E) plotted against the whisker pad crop PC. B: Same as panel A, but using a different crop (in purple) to compute a hand engineered eyelid feature. The PS-VAE unsupervised “Eyelid” latent is highly correlated with this hand engineered feature. C: A 2-state ARHMM fit to the whisker pad PC produces a hand engineered whisker pad movement detector. D: The whisker pad movement detector constructed from the PS-VAE “Whisker pad” latent (reproduced from Fig 5). The continuous signals in panels C, D (and the discrete states derived from them, indicated by the background colors) are similar. E: A confusion matrix shows the overlap between the discrete states inferred from the PS-VAE “Whisker pad” latent and the whisker pad PC (each row adds to 1). F: The discrete states derived from the VAE latents are highly overlapping with those from the whisker pad movement detectors in panels C and D (reproduced from Fig 5). G: Overlap of states derived from the VAE latents and the whisker pad PC show the VAE-based states are highly correlated with whisker movements. (PDF) [file pcbi.1009439.s001.pdf]

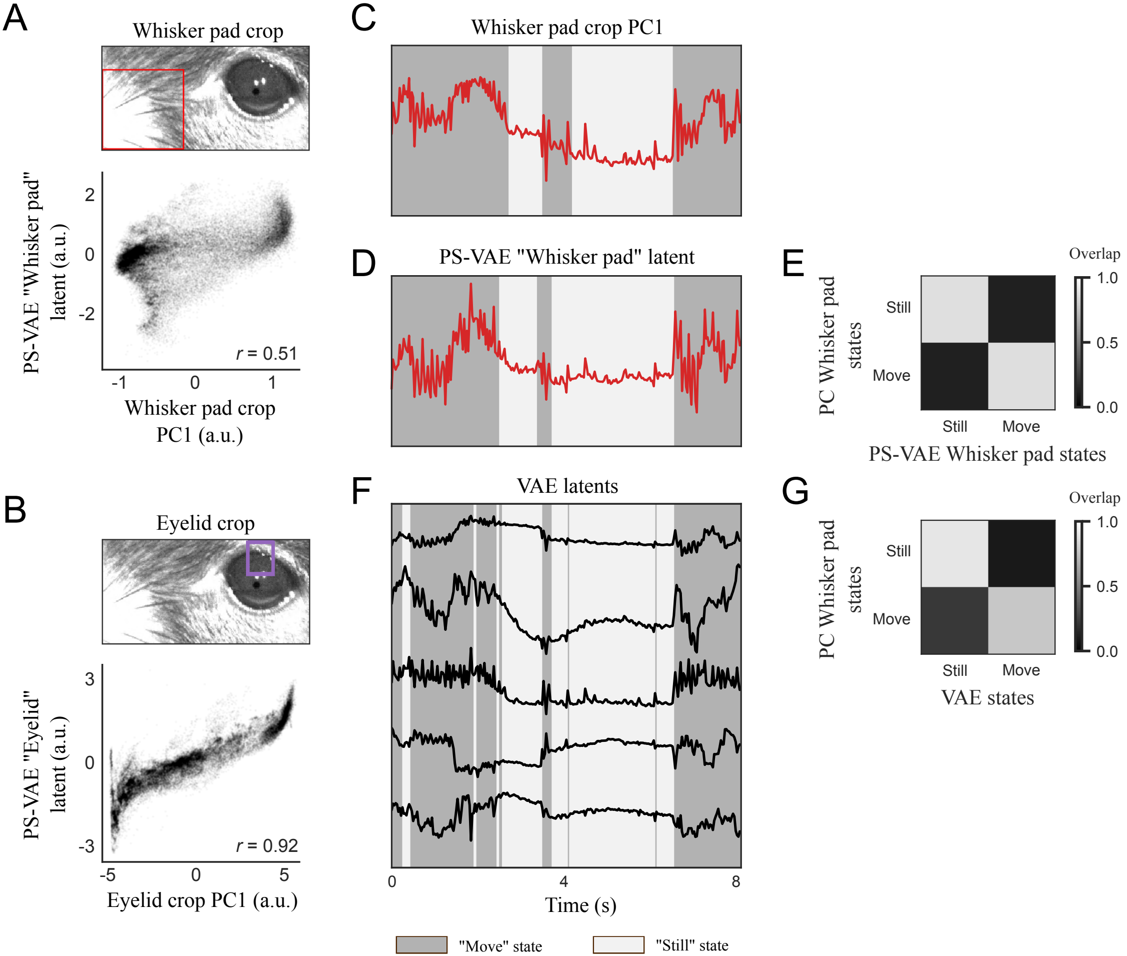
A
Whisker pad crop
PS-VAE "Whisker pad" latent (a.u.)
2
0
-2
r = 0.51
-1
0
1
Whisker pad crop PC1 (a.u.)
B
Eyelid crop
PS-VAE "Eyelid" latent (a.u.)
3
0
-3
r = 0.92
-5
0
5
Eyelid crop PC1 (a.u.)
C
Whisker pad crop PC1
D
PS-VAE "Whisker pad" latent
E
Overlap
1.0
0.5
0.0
PC Whisker pad states
Still
Move
Still
Move
PS-VAE Whisker pad states
F
VAE latents
0
4
8
Time (s)
G
Overlap
1.0
0.5
0.0
PC Whisker pad states
Still
Move
Still
Move
VAE states
"Move" state
"Still" state
