# Supplementary figures and images for: Partitioning variability in animal behavioral videos using semi-supervised variational autoencoders
Source: PLoS Comput Biol. 2021 Sep 22;17(9):e1009439. doi: 10.1371/journal.pcbi.1009439 (PMC8489729; doi:10.1371/journal.pcbi.1009439)

**A**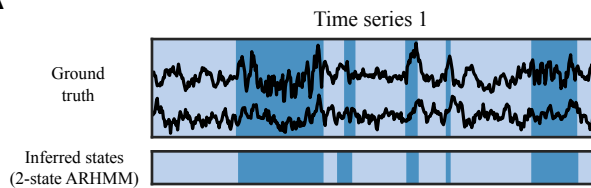**C**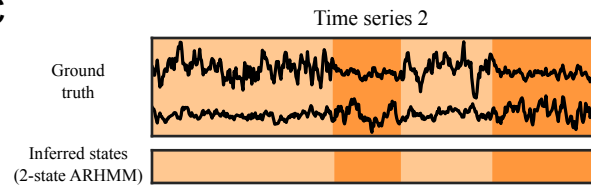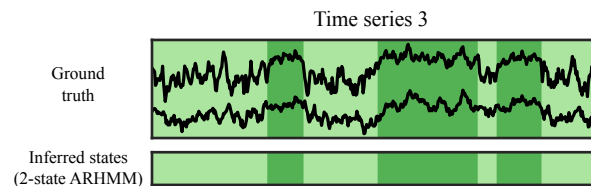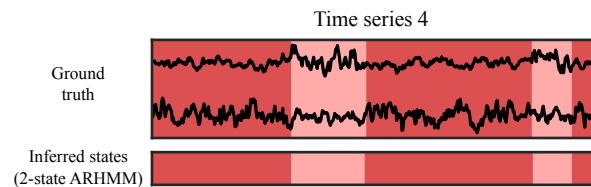**D**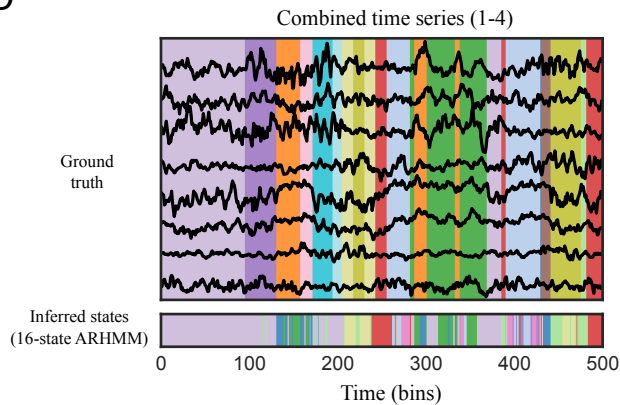**B**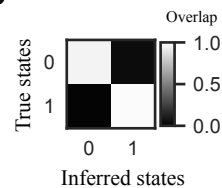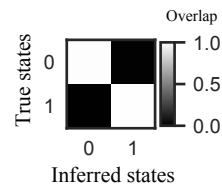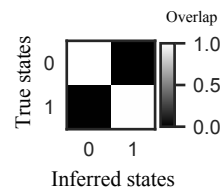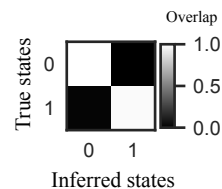**E**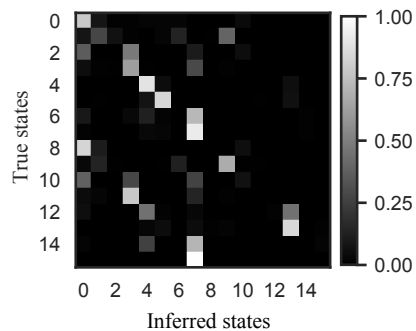

Supplement: S2 Fig — A: Top: A 2D time series is generated using a 2-state ARHMM. The background color indicates the true discrete state at each time point. Bottom: A separate 2-state ARHMM is then fit to this simulated data. The inferred states visually match the true states well on this window of data. B: A confusion matrix shows the overlap between true and inferred states on held-out test data (each row adds to 1). The ARHMM is able to perfectly recover the discrete states. C: This process is repeated three more times to yield four independent time series. In each case an ARHMM is able to perfectly recover the discrete states. D: Top: The four 2D time series from above are stacked to form an 8D time series. This results in data with 24 = 16 discrete states (indicated by the background color), since each of the four independent time series can be in one of two states at each time point. Bottom: A 16-state ARHMM is then fit to this 8D simulated data, resulting in a mismatch between the true and inferred states on some time points. E: The confusion matrix shows many errors in the inferred states due to the larger dimensionality of the data and the larger number of states. By splitting the data into subsets of dimensions as in A and C and fitting a larger number of simple ARHMMs we recover the true discrete states more accurately. (PDF) [file pcbi.1009439.s002.pdf]

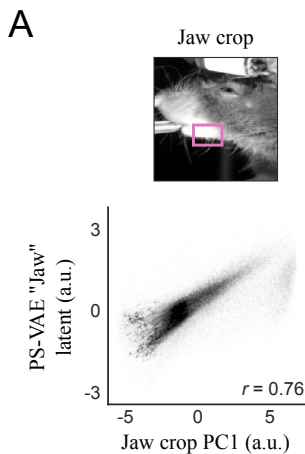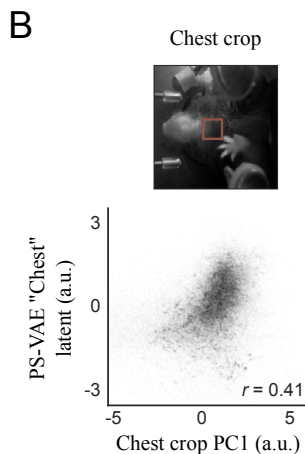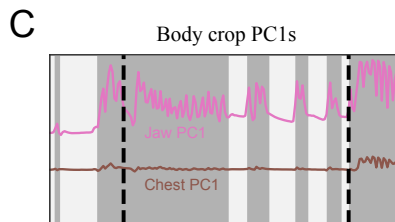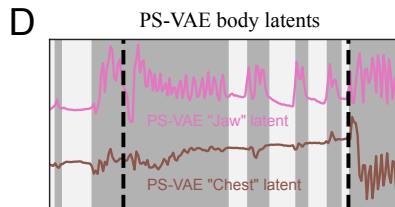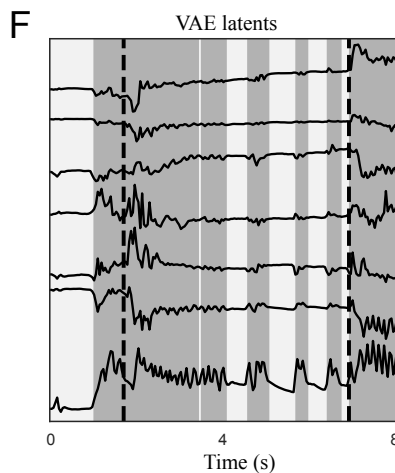

Move state Still state

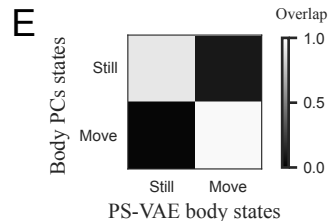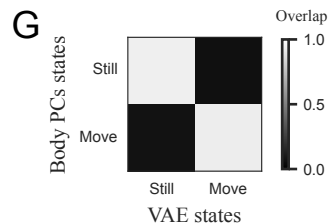

Supplement: S4 Fig — Conventions and conclusions are the same as S1 Fig. A: The pink box outlines the frame crop used to compute a hand engineered jaw feature, which is highly correlated with the PS-VAE unsupervised latent corresponding to the jaw. B: The brown box outlines the crop used to compute a hand engineered chest feature, which is modestly correlated with the PS-VAE unsupervised latent corresponding to the chest. It is difficult to compute such a hand engineered feature that is not contaminated by the paw or the mechanical lever, demonstrating an advantage of the PS-VAE in this dataset. C: A 2-state ARHMM fit to the jaw and chest PCs (the “body” PCs) produces a hand engineered body movement detector. D: The body movement detector constructed from the PS-VAE body latents (reproduced from Fig 8). E: A confusion matrix shows the overlap between the discrete states inferred from the PS-VAE body latents and the body PCs. F: The discrete states derived from the VAE latents are highly overlapping with those from the body movement detectors in panels C and D (reproduced from Fig 8). G: Overlap of states derived from the VAE latents and the body PCs suggests the VAE-based states are highly correlated with body movements. (PDF) [file pcbi.1009439.s004.pdf]

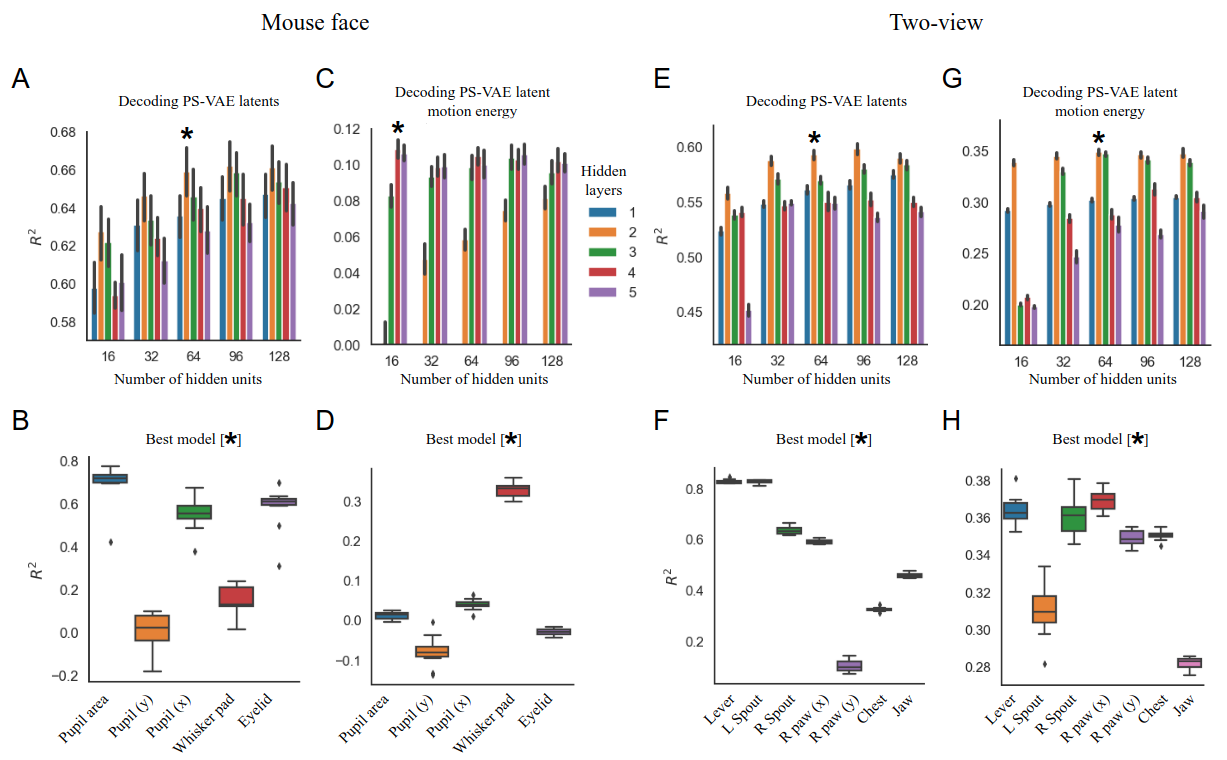

Supplement: S6 Fig — A: Hyperparameter search results for decoding PS-VAE latents from the mouse face data. The “best” model is indicated with an asterisk (*), and is a chosen to balance model performance and model complexity. Error bars represent a 95% bootstrapped confidence interval over 10 random subsamples of 200 neurons. B: R2 results for the best model, separated by latent (reproduced from Fig 6C). Neural activity is able to successfully reconstruct the pupil area, eyelid, and horizontal position of the pupil location. The poor reconstruction of the vertical position of the pupil location may be due to the small dynamic range (and accompanying noise) of that label. The boxplot represents variability in R2 over the 10 random subsamples. C: Hyperparameter search results for decoding PS-VAE latent motion energy (ME) from the mouse face data. Error bars as in A. D: R2 results for the best model, separated by latent. The ME of the whisker pad is decoded well, consistent with the results in [22] and [23]. Boxplot variability as in B. E-H: Same as A-D, except on the two-view dataset. The mechanical equipment (Lever, L Spout, R Spout), which has low trial-to-trial variability (Fig 8A), is decoded better than the animal-related latents. The accuracy of the ME decoding is similar across all latents. Error bars represent a 95% bootstrapped confidence interval over test trials; boxplots represent variability across 10 bootstrapped samples from the test trials (see Methods for more information). (PNG) [file pcbi.1009439.s006.png]

A

PS-VAE latents

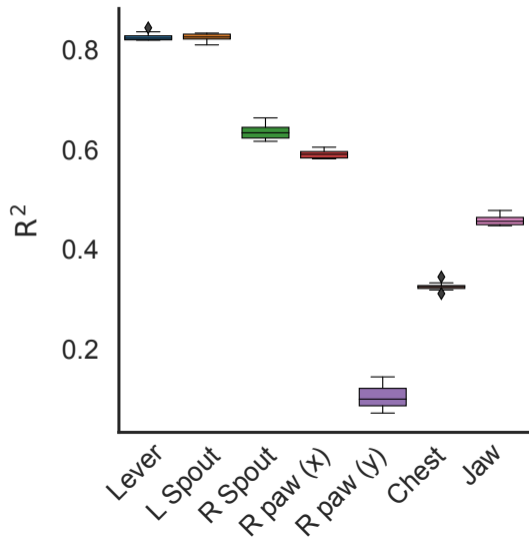

B

VAE latents

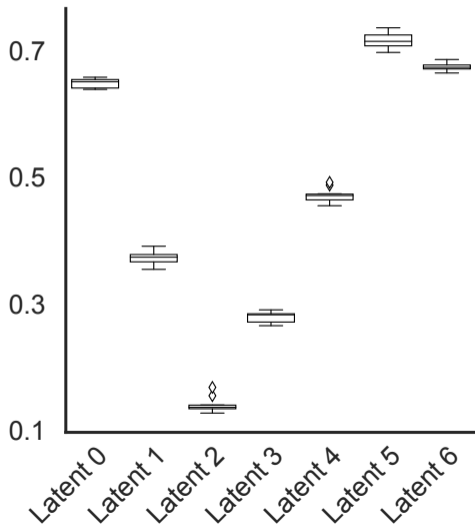

Supplement: S7 Fig — A: Decoding accuracy (R2) computed separately for each PS-VAE latent demonstrates how the PS-VAE can be utilized to investigate the neural representation of different behavioral features (same as S6F Fig). B: Decoding accuracy computed separately for each VAE latent. Boxplots show variability over 10 bootstrapped samples from the test trials (see Methods for more information). (PDF) [file pcbi.1009439.s007.pdf]

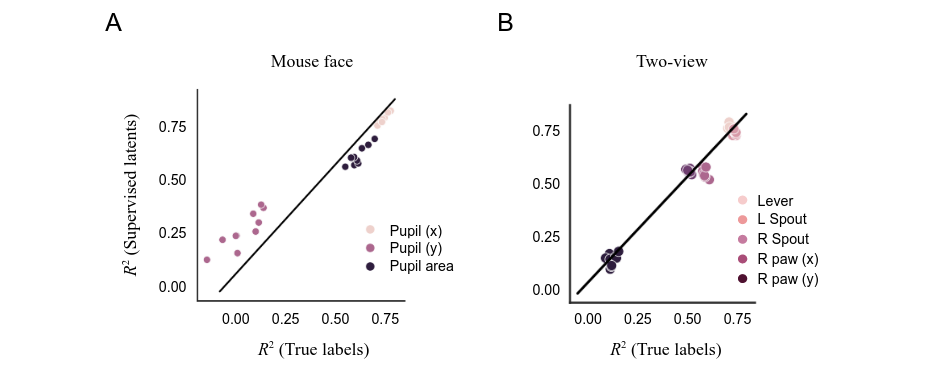

Supplement: S8 Fig — Decoding accuracy for the true labels (x-axis) and their corresponding supervised latents in the PS-VAE (y-axis), for both the mouse face dataset (panel A) and the two-view dataset (panel B); individual dots represent the median over test trials using different subsamples of neurons (panel A) or trials (panel B). The decoding accuracy is very similar across most labels in both datasets, indicating that the noise introduced in the PS-VAE label reconstruction does not have a large effect on the neural decoding of these quantities. The model architecture used for decoding the true labels is the same as the best model architecture found for decoding the PS-VAE latents (S6 Fig). (PNG) [file pcbi.1009439.s008.png]

**A**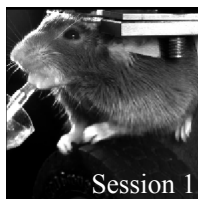

— True  
— MSPS-VAE  
— VAE

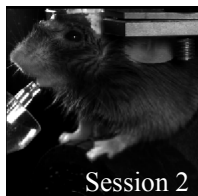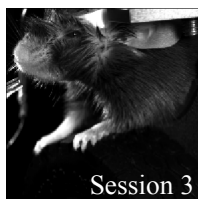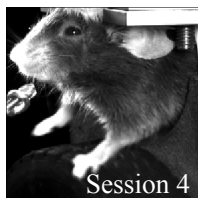**B**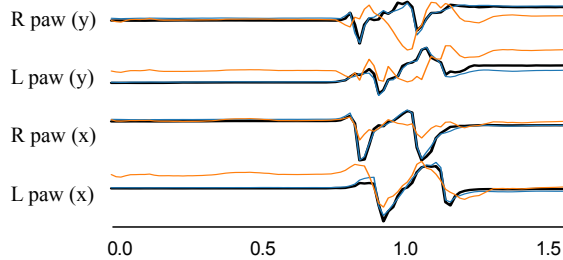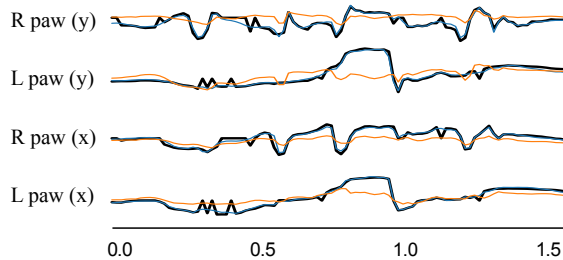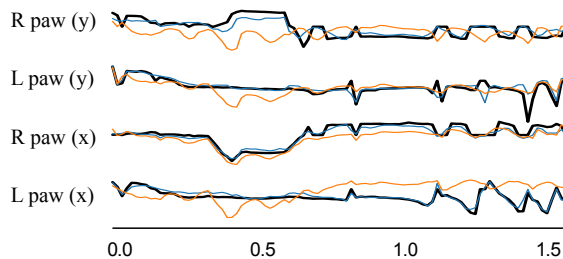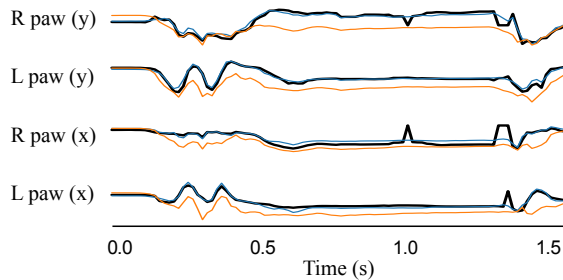**C**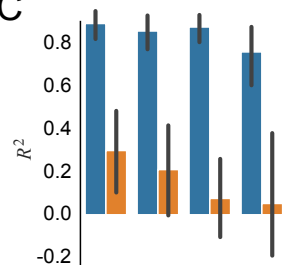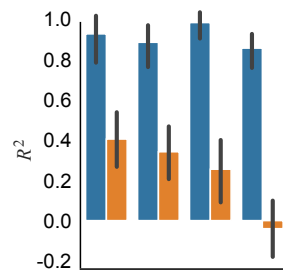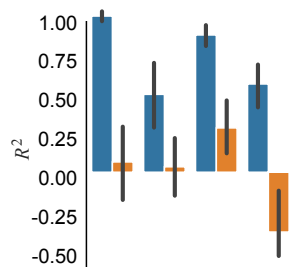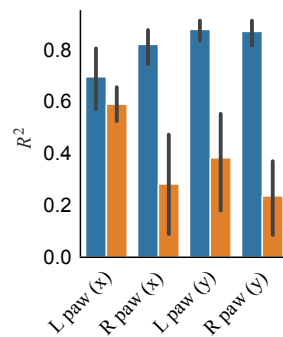

Supplement: S9 Fig — A: Frames from each of the four sessions used to train the models. B: The true labels (black lines) are almost perfectly reconstructed by the supervised subspace of the MSPS-VAE (blue lines). We also reconstruct the labels from the latent representation of a single VAE trained on all sessions (orange lines), which captures some features of the labels but misses much of the variability. C: Observations from the individual batches in panel B hold across all labels and test trials for each sesson. Error bars represent a 95% bootstrapped confidence interval over test trials. (PDF) [file pcbi.1009439.s009.pdf]

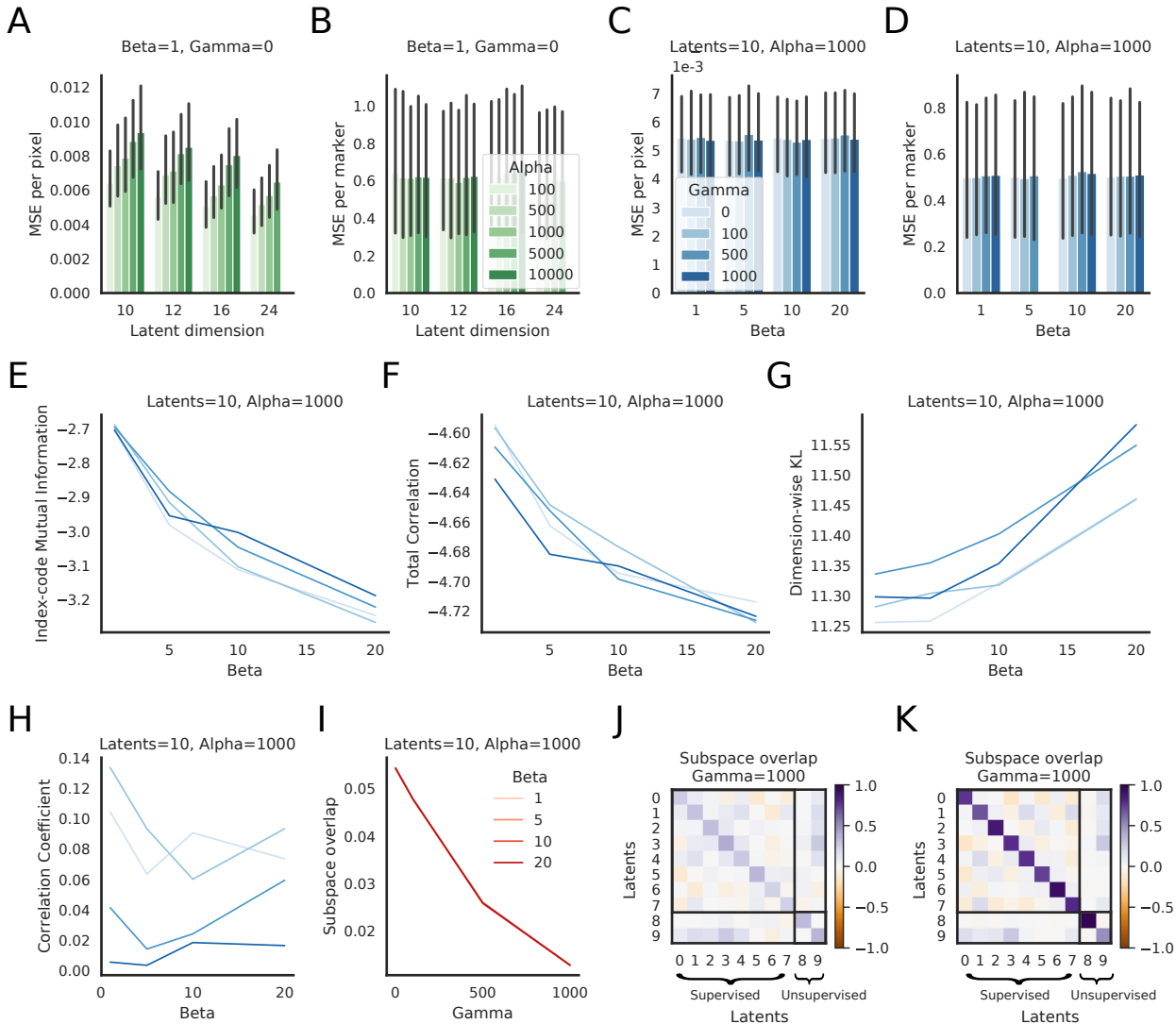

Supplement: S10 Fig — Panel descriptions are the same as those in Fig 11. (PDF) [file pcbi.1009439.s010.pdf]

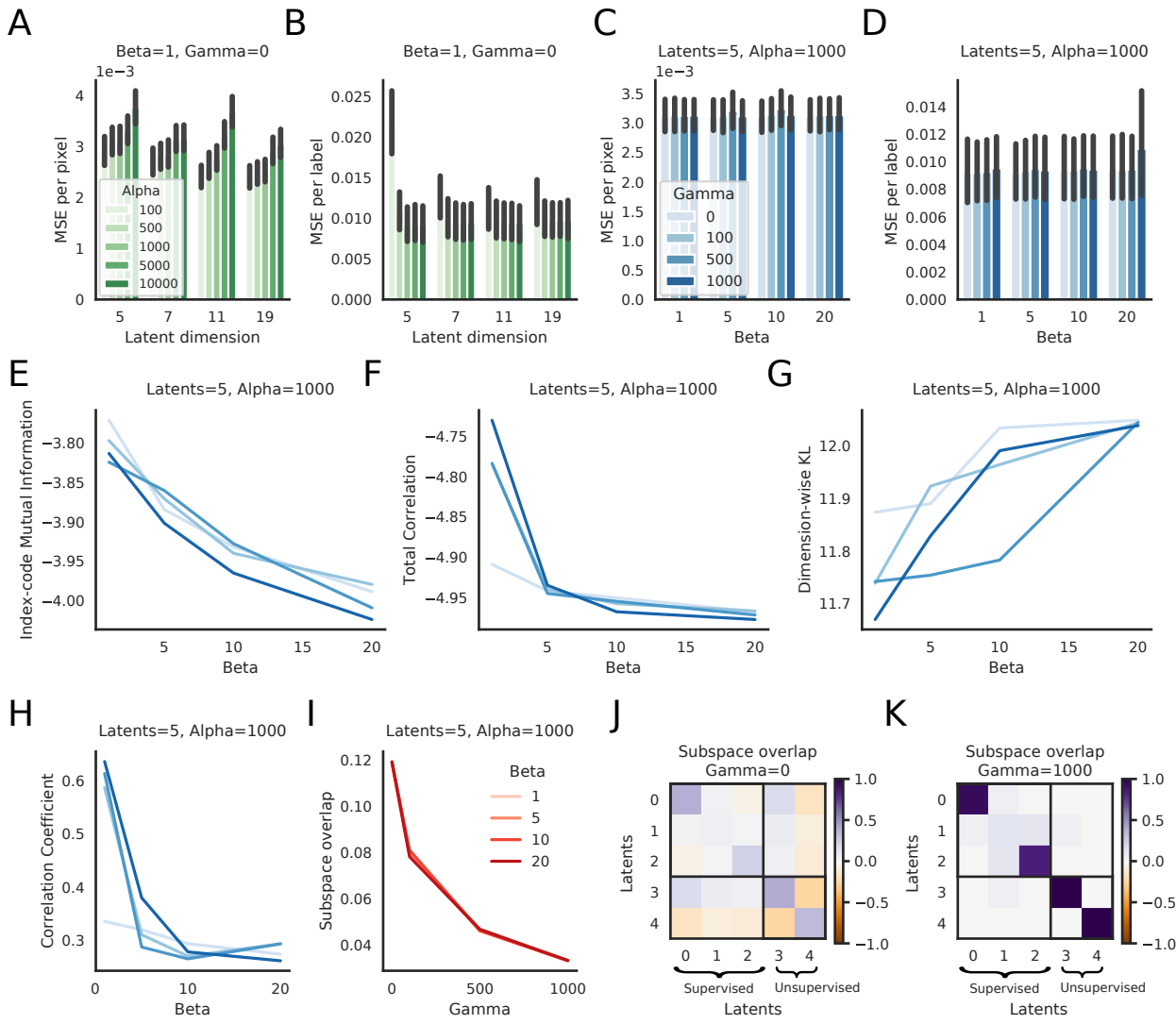

Supplement: S11 Fig — Panel descriptions are the same as those in Fig 11. (PDF) [file pcbi.1009439.s011.pdf]

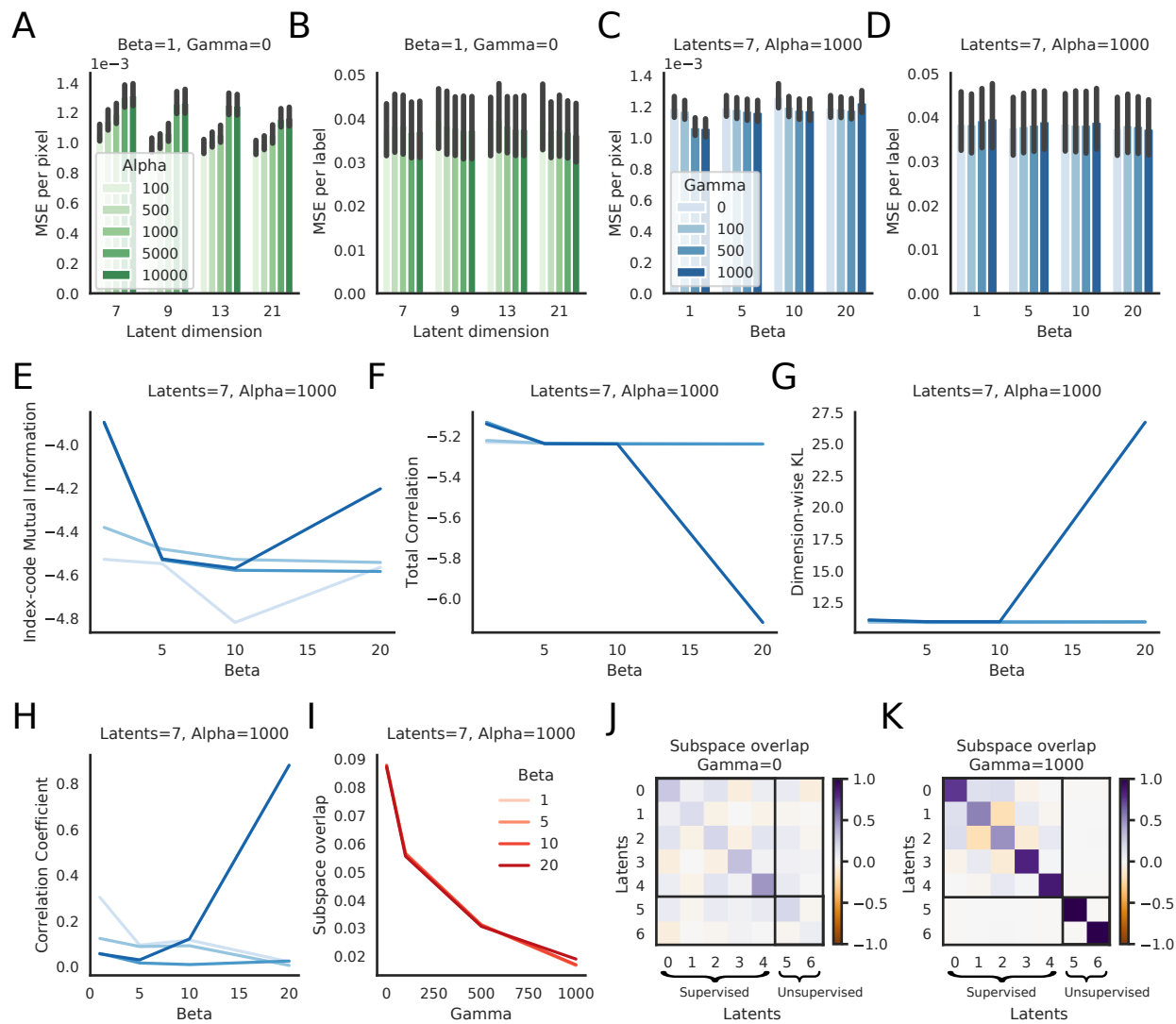

Supplement: S12 Fig — Panel descriptions are the same as those in Fig 11. (PDF) [file pcbi.1009439.s012.pdf]

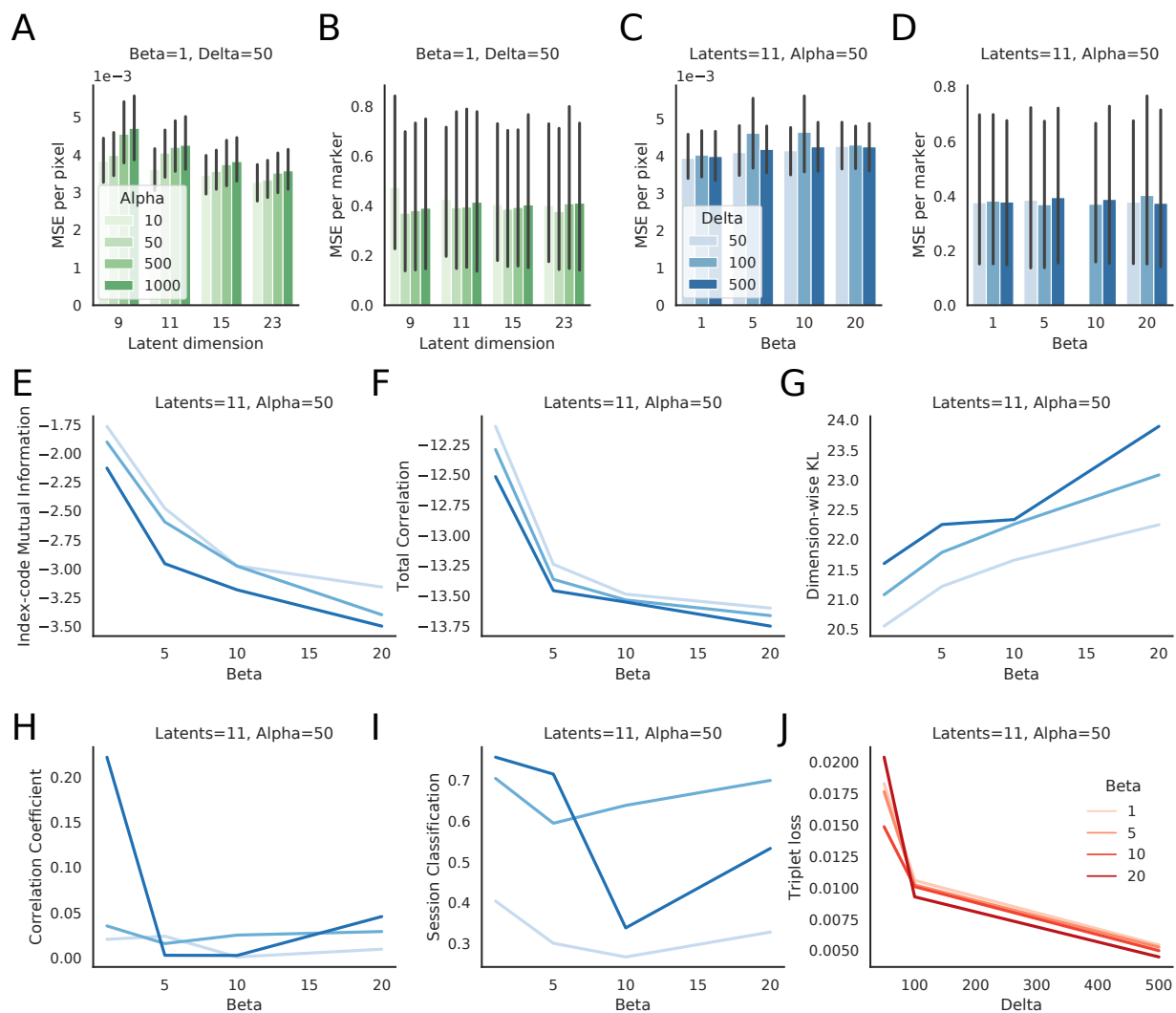

Supplement: S13 Fig — A: MSE per pixel as a function of latent dimensionality and the hyperparameter α, which controls the strength of the label reconstruction term. The frame reconstruction is robust across many orders of magnitude. B: MSE per label as a function of latent dimensionality and α. Subsequent panels detail β and δ with an 11D model (|zs| = 4, |zu| = 4, |zb| = 3) and α = 50. C: MSE per pixel as a function of β and δ; frame reconstruction is robust to both of these hyperprameters. D: MSE per label as a function of β and δ; label reconstruction is robust to both of these hyperprameters. E: Index code mutual information as a function of β and δ. F: Total Correlation as a function of β and δ. G: Dimension-wise KL as a function of β and δ. H: Average of all pairwise Pearson correlation coefficients in the model’s 4D unsupervised subspace as a function of β and δ. I: Session classification accuracy from a linear classifier that predicts session identity from the unsupervised latents, as a function of β and δ. J: Triplet loss as a function of β and δ. Error bars in panels A-D represent 95% bootstrapped confidence interval over test trials; line plots in panels E-J are the mean values over test trials, and confidence intervals are omitted for clarity. (PDF) [file pcbi.1009439.s013.pdf]

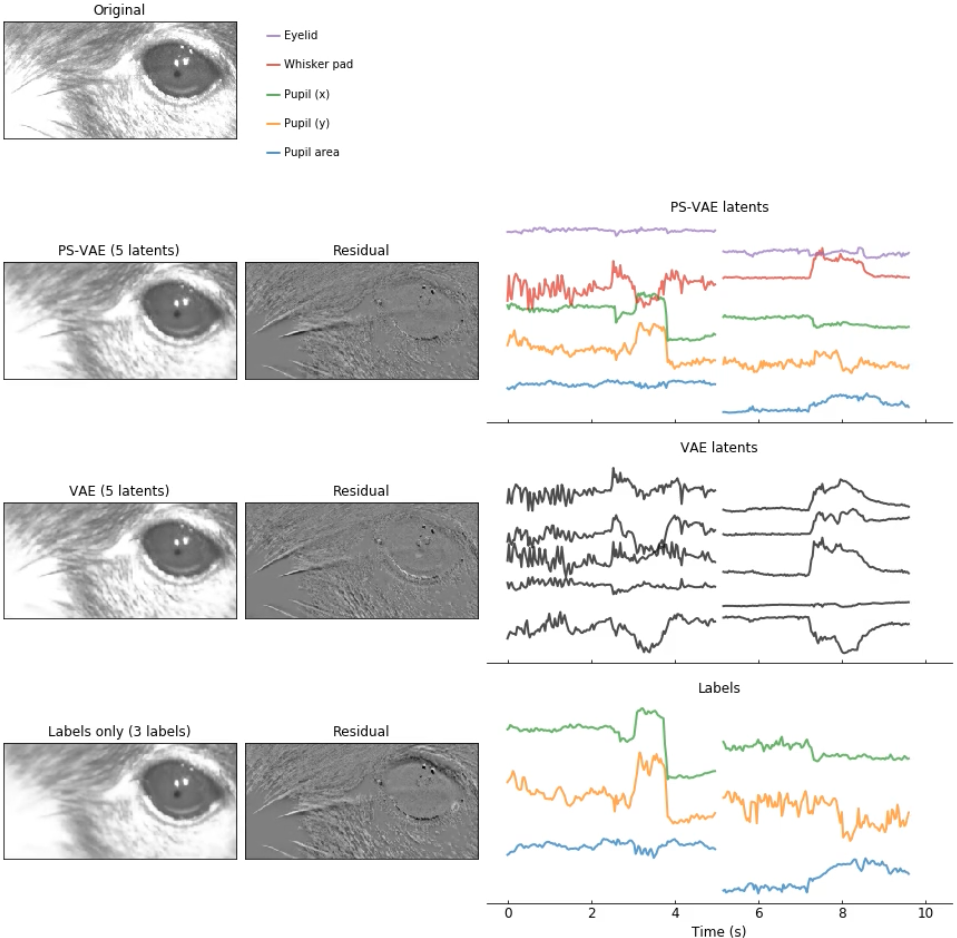

Supplement: S14 Fig — First (top) row: frames from the original behavioral video. Second row: reconstructed frames from the PS-VAE (left), residuals between these reconstructed frames and the original frames (center), and the corresponding PS-VAE latents (right). Colors are consistent with those in the main figures, and latent names are provided in the legend above. Third row: reconstructed frames from a standard VAE, the residuals, and the corresponding VAE latents. Fourth row: frames reconstructed from the labels only, without unsupervised latent dimensions (again with the residuals and the corresponding true labels). Reconstructions are shown for several batches of test data not used for training or model selection. Each batch is separated by black frames in the reconstructions and breaks in the traces. (PNG) [file pcbi.1009439.s014.png]

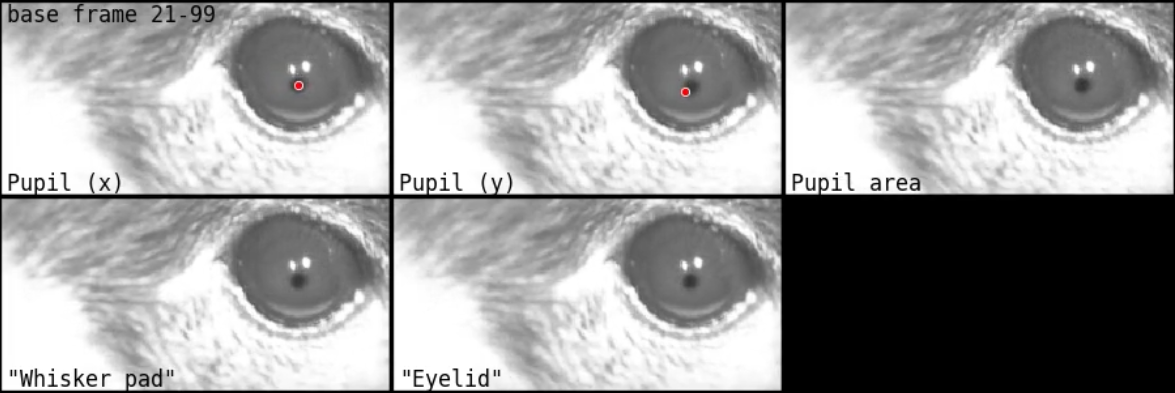

Supplement: S15 Fig — Each panel contains latent traversals for a single dimension, the name of which is indicated in the lower left corner of the panel. Supervised latents with associated labels use the label names; unsupervised latents that we have applied a post-hoc semantic label to are indicated with quotations, such as “Whisker pad” and “Eyelid” here. Unsupervised latents that have not been given labels (for example the VAE latents) are named Latent 0, Latent 1, etc. (none of this type are shown here). The supervised dimensions that correspond to 2D spatial locations contain an additional red dot that signifies the desired position of the body part, seen here in the Pupil (x) and Pupil (y) panels. A missing dot indicates a low-likelihood label that was omitted from the objective function. The latent traversal procedure uses a base frame, indicated in the upper left corner of the figure. The videos show traversals for a range of base frames, and traversals for different base frames are separated by several black frames. (PNG) [file pcbi.1009439.s015.png]

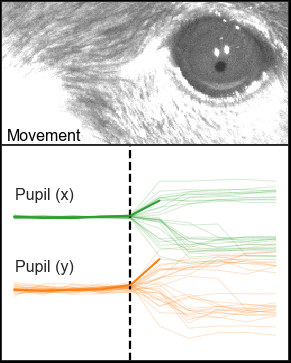

Supplement: S16 Fig — Top: examples of video are played from 5 frames before to 5 frames after the transition from the still state to the moving state, as identified by the ARHMM. The state for each frame is indicated by the text in the bottom left corner. Different examples are separated by several black frames. Bottom: Corresponding traces of the PS-VAE latents used to fit the ARHMM; the latents corresponding to the current example are displayed in bold. (PNG) [file pcbi.1009439.s016.png]

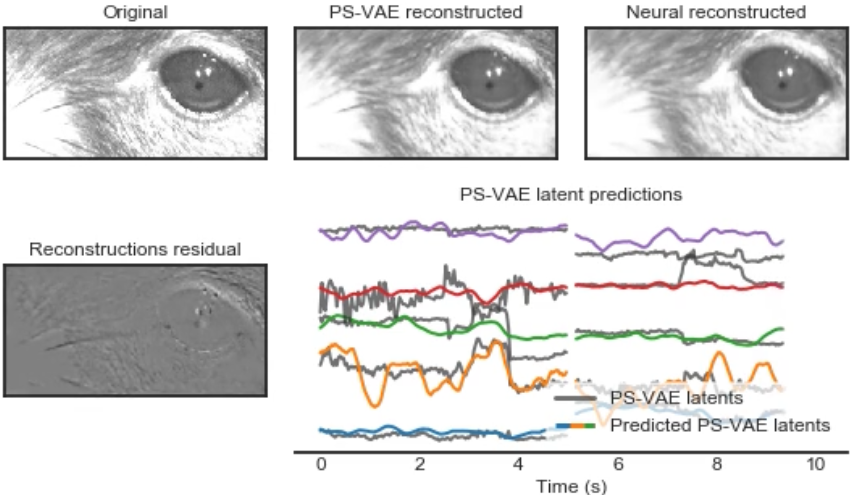

Supplement: S17 Fig — Top left: frames from the original behavioral video. Top center: reconstructed frames from the PS-VAE. Top right: frames reconstructed by the neural activity. Bottom left: residual between the PS-VAE and neural reconstructions. Bottom right: PS-VAE latents (gray traces) and their predictions from neural activity (colored traces; colors are consistent with those in the main figures). Reconstructions are shown for several batches of test data, which were not used for the training or model selection of either the PS-VAE or the neural decoders. Each test batch is separated by black frames in the frame reconstructions, and breaks in the traces. (PNG) [file pcbi.1009439.s017.png]

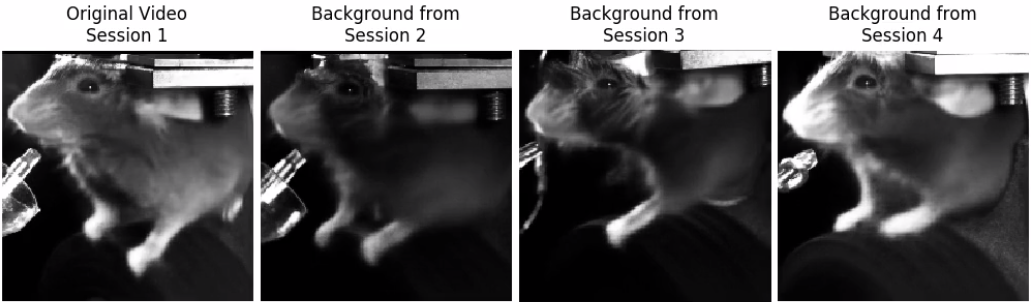

Supplement: S18 Fig — On the left are MSPS-VAE reconstructions from the original behavioral video. Each remaining panel shows frames reconstructed from the same set of latents, except the background latents have been set to the median value of the background latents for the indicated session. (PNG) [file pcbi.1009439.s018.png]

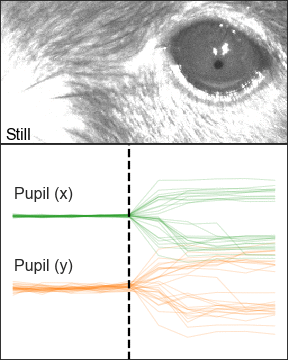

Supplement: S21 Video — Refer to S16 Fig for captions. (GIF) [file pcbi.1009439.s039.gif]

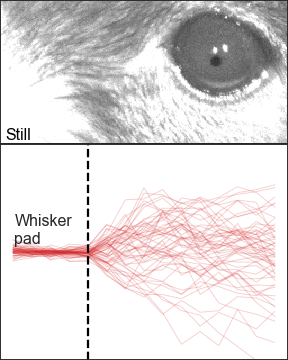

Supplement: S22 Video — Refer to S16 Fig for captions. (GIF) [file pcbi.1009439.s040.gif]

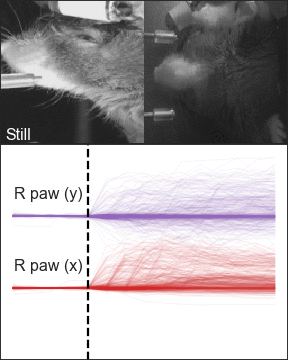

Supplement: S23 Video — Refer to S16 Fig for captions. (GIF) [file pcbi.1009439.s041.gif]

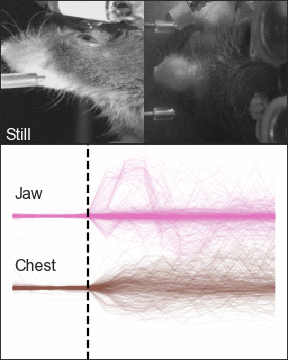

Supplement: S24 Video — Refer to S16 Fig for captions. (GIF) [file pcbi.1009439.s042.gif]
