# Supplementary material for: Partitioning variability in animal behavioral videos using semi-supervised variational autoencoders
Source: PLoS Comput Biol. 2021 Sep 22;17(9):e1009439. doi: 10.1371/journal.pcbi.1009439 (PMC8489729; doi:10.1371/journal.pcbi.1009439)
Supplement: S3 Fig — A: [Reproduced from Fig 5.] Top: Supervised PS-VAE latents corresponding to pupil location. Background colors indicate the states recovered by the 2-state saccade detector, which we call “pupil still” (light gray) and “pupil move” (dark gray). Bottom: Unsupervised PS-VAE latent corresponding to the whisker pad, and the states recovered by the 2-state whisking detector—“whisk still” (light gray) and “whisk move” (dark gray). B: The states from panel A combinatorially define four unique states (since each of the two ARHMMs can be in one of two states at each time point), which we refer to as the “combinatorial” PS-VAE states. C: The pupil location and whisker pad latents are concatenated and fit with a 4-state ARHMM. We refer to the resulting states as the “combined” PS-VAE states. There is general agreement between the combined and combinatorial states, although the combined states contain more state switches. D: A confusion matrix shows the overlap between between the combinatorial and combined states across all held-out test data. There remain many incongruous time points—for example, only 61% of the time points identified by the combinatorial state “pupil move/whisk move” is captured in a single combined state. E: A 4-state ARHMM is fit to the VAE latents. The resulting segmentation is somewhat aligned with the combinatorial PS-VAE segmentation in panel B but is much noisier, especially during whisker movements. F: There is poor overlap between the combinatorial PS-VAE states and the VAE states, suggesting that the VAE states are not capturing simple combinations of pupil and whisker movement. However, due to the lack of interpretability in the VAE latents, it is difficult to assess from this visualization alone what behaviors the VAE states capture. (PDF) [file pcbi.1009439.s003.pdf]

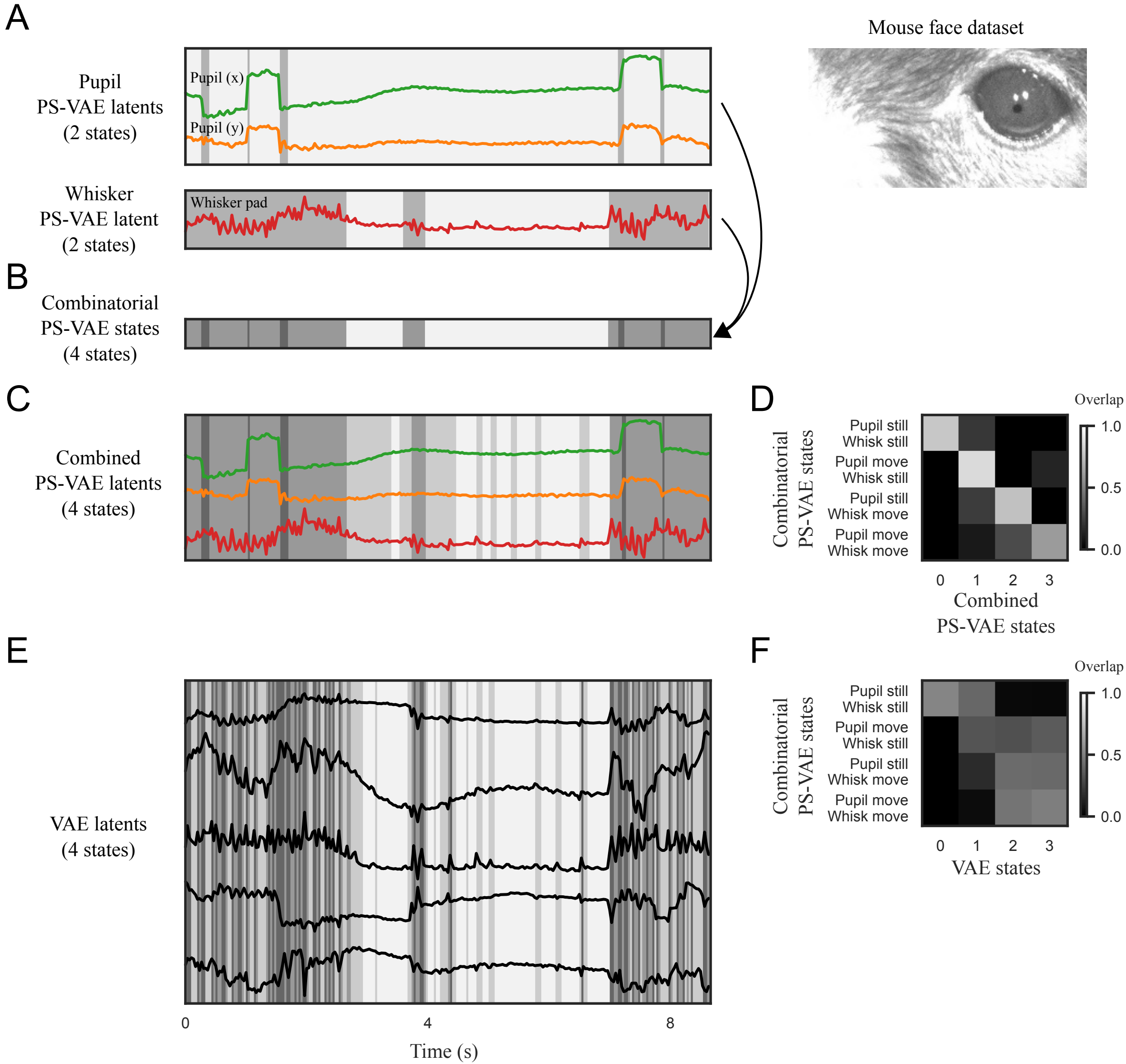

A
Mouse face dataset
Pupil
PS-VAE latents
(2 states)
Pupil (x)
Pupil (y)
Whisker
PS-VAE latent
(2 states)
Whisker pad
B
Combinatorial
PS-VAE states
(4 states)
C
Combined
PS-VAE latents
(4 states)
D
Overlap
Combinatorial
PS-VAE states
Pupil still
Whisk still
Pupil move
Whisk still
Pupil still
Whisk move
Pupil move
Whisk move
1.0
0.5
0.0
0 1 2 3
Combined
PS-VAE states
E
VAE latents
(4 states)
0
4
8
Time (s)
F
Overlap
Combinatorial
PS-VAE states
Pupil still
Whisk still
Pupil move
Whisk still
Pupil still
Whisk move
Pupil move
Whisk move
1.0
0.5
0.0
0 1 2 3
VAE states
