# Supplementary material for: Partitioning variability in animal behavioral videos using semi-supervised variational autoencoders
Source: PLoS Comput Biol. 2021 Sep 22;17(9):e1009439. doi: 10.1371/journal.pcbi.1009439 (PMC8489729; doi:10.1371/journal.pcbi.1009439)
Supplement: S5 Fig — Conventions and conclusions are the same as S3 Fig. A: [Reproduced from Fig 8.] Top: Supervised PS-VAE latents corresponding to paw location. Bottom: Unsupervised PS-VAE latents corresponding to the body. B: The states from panel A combinatorially define four unique states. C: The paw and body latents are concatenated and fit with a 4-state ARHMM. There is general agreement between the combined and combinatorial states, although the combined states contain more state switches. D: A confusion matrix shows the overlap between between the combinatorial and combined states across all held-out test data. There remain many incongruous time points—for example, only 63% of the time points identified by the combinatorial state “paw move/body move” is captured in a single combined state. E: A 4-state ARHMM is fit to the VAE latents. The resulting segmentation is well aligned with the combinatorial PS-VAE segmentation in panel B, but tends to be noisier during body movements. F: There is poor overlap between the combinatorial PS-VAE states and the VAE states, suggesting that the VAE states are not capturing simple combinations of paw and body movements. (PDF) [file pcbi.1009439.s005.pdf]

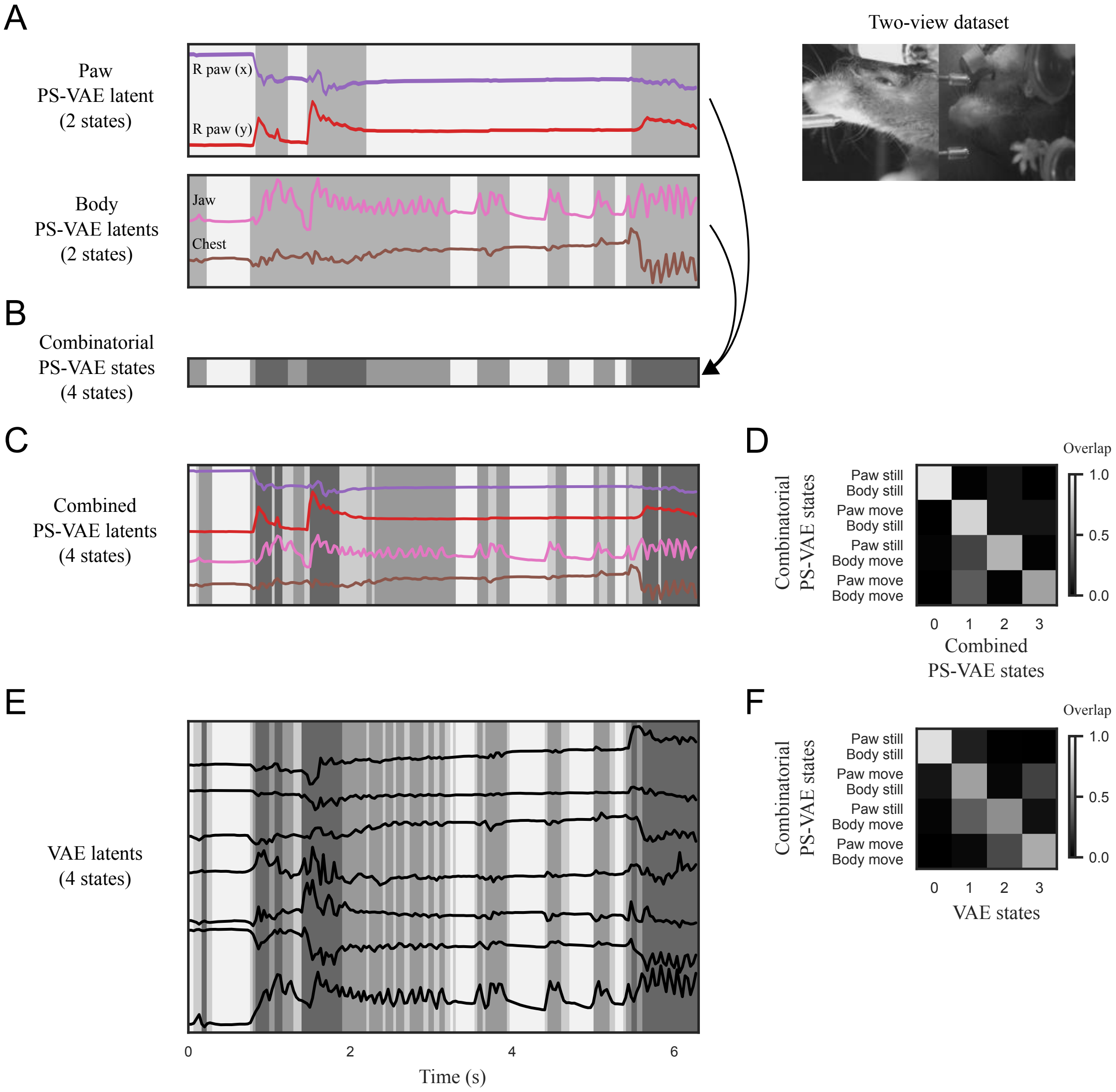

A
Two-view dataset
Paw
PS-VAE latent
(2 states)
R paw (x)
R paw (y)
Body
PS-VAE latents
(2 states)
Jaw
Chest
B
Combinatorial
PS-VAE states
(4 states)
C
Combined
PS-VAE latents
(4 states)
D
Overlap
1.0
0.5
0.0
Paw still
Body still
Paw move
Body still
Paw still
Body move
Paw move
Body move
Combinatorial
PS-VAE states
0
1
2
3
Combined
PS-VAE states
E
VAE latents
(4 states)
0
2
4
6
Time (s)
F
Overlap
1.0
0.5
0.0
Paw still
Body still
Paw move
Body still
Paw still
Body move
Paw move
Body move
Combinatorial
PS-VAE states
0
1
2
3
VAE states
