# Supplementary material for: Partitioning variability in animal behavioral videos using semi-supervised variational autoencoders
Source: PLoS Comput Biol. 2021 Sep 22;17(9):e1009439. doi: 10.1371/journal.pcbi.1009439 (PMC8489729; doi:10.1371/journal.pcbi.1009439)
Supplement: S1 Appendix — (PDF) [file pcbi.1009439.s046.pdf]

Related Work

In this appendix, we contextualize the PS-VAE within the broader generative modeling literature. Since the advent of variational autoencoders (VAEs; Kingma and Welling (2013), Rezende et al. (2014), and Titsias et al. (2014)), a growing body of work has established strategies to manipulate the latent variables to make them more interpretable. We divide the VAE variants into two classes: those which are agnostic to the labels (unsupervised latent control), and those which use them (supervised latent control). For a more complete review of VAE variants we refer the reader to Kingma and Welling (2019).

1 Unsupervised latent control

Information theoretic approaches. A natural way to control the latent variables is to place information-theoretic penalties on their properties, without necessarily modifying their prior distribution. Chen et al. (2018) decomposed the KL divergence term in the VAE objective (Eq 9) to a sum of three terms: ⓘ mutual information between the inputs (typically images) and their latent representation; (ii) mutual information between the latent factors, quantifying statistical dependence between them (a.k.a. "Total Correlation"); and (iii) KL divergence between each latent factor and its prior. Chen et al. (2018) then show that overweighting (ii) results in strong disentanglement: traversing each dimension of the latent vector results in unique semantic change in the image (see Kim et al. (2018) for a different approach to the same idea). This model (the β-TC-VAE) corresponds to the unsupervised latent space in our model, except that we also encourage those latent factors to be orthogonal to each other, resembling PCA.

Factorized Priors. A second family of models enforces structure in the latents' prior. It has been shown that building inductive biases into the priors leads to better representations and better reconstructions (Mathieu et al. 2019). Instead of assuming independent standard normal factors, we may encourage the multivariate latent distribution to capture a range of structures: to cluster (Mathieu et al. 2019; Dilokthanakul et al. 2017; Graving et al. 2020)), to include arbitrarily rich correlations between each pair of latents (Casale et al. 2018), to follow temporal dynamics (Johnson et al. 2016; Gao et al. 2019; Krishnan et al. 2015), or to be sparse (Mathieu et al. 2019). Our model assumes the simplest standard normal prior but can easily accommodate more structured priors for different applications. We leave this extension for future work.

2 Supervised latent control.

This class of models uses both the images and the labels to influence the latent vector. We subdivide it further into *discriminative* approaches that use a discriminative (i.e. regression) model to predict the labels from the latent representation, and *conditional* approaches that explicitly condition the latent representation on the labels.

Discriminative approaches. The PS-VAE is a discriminative approach: regressing the latents onto the labels allows us to explicitly treat the observation noise in our labels, which is often non-negligble in pose estimation algorithms. Other discriminative algorithms have been proposed that incorporate labels in various ways (Yu et al. 2006; Zhuang et al. 2015; Gogna et al. 2016; Pu et al. 2016; Tissera et al. 2016; Le et al. 2018; Miller et al. 2019; Zheng et al. 2019; Li et al. 2020). We take our inspiration from Li et al. (2020), which explicitly partitions the latent space into orthogonal supervised (label-relevant) and unsupervised (label-irrelevant) subspaces; however, this model does not attempt to disentangle the unsupervised latent space, as we do by penalizing Total Correlation.

Conditional approaches. A straightforward yet influential conditional approach is the conditional VAE (Kingma, Mohamed, et al. 2014; Sohn et al. 2015), which simply appends the labels to the latent vector, making them available for the decoder to use for image reconstruction. However, we have found this approach to be sensitive to noise in the labels (Wu et al. 2020). Other recent work has focused on modeling the distributions of the latents conditioned on the labels (Khemakhem et al. 2020; Zhou et al. 2020). This strategy can also lead to disentangled latents, but it does not produce separate label-relevant and -irrelevant subspaces.

The PS-VAE is a novel synthesis of supervised and unsupervised latent control algorithms. Its latent space is partitioned into orthogonal supervised and unsupervised subspaces based on labels (Li et al. 2020). Through information theoretic and linear algebraic constraints, the PS-VAE's novel unsupervised subspace comprises independent and orthogonal factors (Chen et al. 2018; Kim et al. 2018), which is crucial for downstream scientific analyses.

References

[1] Diederik P Kingma and Max Welling. "Auto-encoding variational bayes." *arXiv preprint arXiv:1312.6114* (2013).

[2] Danilo Jimenez Rezende, Shakir Mohamed, and Daan Wierstra. "Stochastic backpropagation and approximate inference in deep generative models." *arXiv preprint arXiv:1401.4082* (2014).

[3] Michalis Titsias and Miguel Lázaro-Gredilla. "Doubly stochastic variational Bayes for non-conjugate inference." *International Conference on Machine Learning*. 2014, pp. 1971–1979.

[4] Diederik P Kingma and Max Welling. "An introduction to variational autoencoders." *arXiv preprint arXiv:1906.02691* (2019).

[5] Ricky TQ Chen et al. "Isolating sources of disentanglement in variational autoencoders." *Advances in Neural Information Processing Systems*. 2018, pp. 2610–2620.

[6] Hyunjik Kim and Andriy Mnih. "Disentangling by factorising." *arXiv preprint arXiv:1802.05983* (2018).

[7] Emile Mathieu et al. "Disentangling Disentanglement in Variational Autoencoders." *Proceedings of the 36th International Conference on Machine Learning*. Ed. by Kamalika Chaudhuri and Ruslan Salakhutdinov. Vol. 97. Proceedings of Machine Learning Research. PMLR, Sept. 2019, pp. 4402–4412. URL: http://proceedings.mlr.press/v97/mathieu19a.html.

[8] Nat Dilokthanakul et al. *Deep Unsupervised Clustering with Gaussian Mixture Variational Autoencoders*. 2017. arXiv: 1611.02648 [cs.LG].

[9] Jacob M Graving and Iain D Couzin. "VAE-SNE: a deep generative model for simultaneous dimensionality reduction and clustering." *BioRxiv* (2020).

[10] Francesco Paolo Casale et al. "Gaussian process prior variational autoencoders." *arXiv preprint arXiv:1810.11738* (2018).

[11] Matthew Johnson et al. "Composing graphical models with neural networks for structured representations and fast inference." *Advances in Neural Information Processing Systems*. 2016, pp. 2946–2954.

[12] Shuyang Gao et al. "Auto-encoding total correlation explanation." *The 22nd International Conference on Artificial Intelligence and Statistics*. 2019, pp. 1157–1166.

[13] Rahul G Krishnan, Uri Shalit, and David Sontag. "Deep kalman filters." *arXiv preprint arXiv:1511.05121* (2015).

[14] Shipeng Yu et al. "Supervised probabilistic principal component analysis." *Proceedings of the 12th ACM SIGKDD international conference on Knowledge discovery and data mining*. 2006, pp. 464–473.

[15] Fuzhen Zhuang et al. "Supervised representation learning: Transfer learning with deep autoencoders." *Twenty-Fourth International Joint Conference on Artificial Intelligence*. 2015.

[16] Anupriya Gogna and Angshul Majumdar. "Semi supervised autoencoder." *International Conference on Neural Information Processing*. Springer. 2016, pp. 82–89.

[17] Yunchen Pu et al. "Variational autoencoder for deep learning of images, labels and captions." *Advances in Neural Information Processing Systems*. 2016, pp. 2352–2360.

[18] Migel D Tissera and Mark D McDonnell. "Deep extreme learning machines: supervised autoencoding architecture for classification." *Neurocomputing* 174 (2016), pp. 42–49.

[19] Lei Le, Andrew Patterson, and Martha White. "Supervised autoencoders: Improving generalization performance with unsupervised regularizers." *Advances in Neural Information Processing Systems*. 2018, pp. 107–117.

[20] Andrew Miller et al. "Discriminative Regularization for Latent Variable Models with Applications to Electrocardiography." *International Conference on Machine Learning*. 2019, pp. 4585–4594.

[21] Zhilin Zheng and Li Sun. "Disentangling latent space for vae by label relevant/irrelevant dimensions." *Proceedings of the IEEE/CVF Conference on Computer Vision and Pattern Recognition*. 2019, pp. 12192–12201.

[22] Xiao Li et al. "Latent space factorisation and manipulation via matrix subspace projection." *International Conference on Machine Learning*. PMLR. 2020, pp. 5916–5926.

[23] Durk P Kingma, Shakir Mohamed, et al. "Semi-supervised learning with deep generative models." *Advances in Neural Information Processing Systems*. 2014, pp. 3581–3589.

[24] Kihyuk Sohn, Honglak Lee, and Xinchen Yan. "Learning structured output representation using deep conditional generative models." *Advances in Neural Information Processing Systems*. 2015, pp. 3483–3491.

[25] Anqi Wu et al. "Deep Graph Pose: a semi-supervised deep graphical model for improved animal pose tracking." *Advances in Neural Information Processing Systems*. 2020, pp. 6040–6052.

[26] Ilyes Khemakhem et al. "Variational autoencoders and nonlinear ica: A unifying framework." *International Conference on Artificial Intelligence and Statistics*. 2020, pp. 2207–2217.

[27] Ding Zhou and Xue-Xin Wei. "Learning identifiable and interpretable latent models of high-dimensional neural activity using pi-VAE." *Advances in Neural Information Processing Systems* 33 (2020).
