# Supplementary material for: Partitioning variability in animal behavioral videos using semi-supervised variational autoencoders
Source: PLoS Comput Biol. 2021 Sep 22;17(9):e1009439. doi: 10.1371/journal.pcbi.1009439 (PMC8489729; doi:10.1371/journal.pcbi.1009439)
Supplement: S1 Table — |zs|, |zu|, and |zb| denote the dimensionality of the supervised, unsupervised, and background latent spaces. *We used the orthogonalization procedure outlined in the MSPS-VAE Methods section to remove γ as a hyperparameter for this model. (PDF) [file pcbi.1009439.s047.pdf]

Figure	Panels	Dataset	Model	$\lvert\mathbf{z}_s\rvert$	$\lvert\mathbf{z}_u\rvert$	$\lvert\mathbf{z}_b\rvert$	α	β	γ	δ
2	A-H	head-fixed mouse	PS-VAE	4	2	0	1000	5	500	-
2	C-D	head-fixed mouse	VAE	0	6	0	-	1	-	-
3	B-E	freely moving mouse	PS-VAE	8	2	0	100	5	1000	-
3	B-C	freely moving mouse	VAE	0	10	0	-	1	-	-
4	B-E	mouse face	PS-VAE	3	2	0	1000	20	1000	-
4	B-C	mouse face	VAE	0	5	0	-	1	-	-
5	A-D	mouse face	PS-VAE	3	2	0	1000	20	1000	-
5	E-G	mouse face	VAE	0	5	0	-	1	-	-
6	B-C	mouse face	PS-VAE	3	2	0	1000	20	1000	-
6	D-E	mouse face	VAE	0	5	0	-	1	-	-
7	B-E	two-view mouse	PS-VAE	5	2	0	1000	1	1000	-
7	B-C	two-view mouse	VAE	0	7	0	-	1	-	-
8	A-C, E-F	two-view mouse	PS-VAE	5	2	0	1000	1	1000	-
8	A, D-F	two-view mouse	VAE	0	7	0	-	1	-	-
9	A-B	two-view mouse	PS-VAE	5	2	0	1000	1	1000	-
9	C	two-view mouse	VAE	0	7	0	-	1	-	-
10	D-G	multi head-fixed mouse	MSPS-VAE	4	4	3	50	10	-	50
10	C	multi head-fixed mouse	PS-VAE	4	7	0	50	10	-*	-
10	B	multi head-fixed mouse	VAE	0	11	0	-	1	-	-
