# Supplementary material for: Partitioning variability in animal behavioral videos using semi-supervised variational autoencoders
Source: PLoS Comput Biol. 2021 Sep 22;17(9):e1009439. doi: 10.1371/journal.pcbi.1009439 (PMC8489729; doi:10.1371/journal.pcbi.1009439)
Supplement: Attachment — Submitted filename: psvae_review_final.pdf [file pcbi.1009439.s048.pdf]

The world is hungry for new behavioral measurement and insight extraction techniques. To address this, the authors introduce a new dimensionality reduction tool, the PS-VAE (Partitioned Subspace Variational Autoencoder) that offers the ability to produce interpretable latent factors describing behavioral video data that additionally possesses some hand labeled keypoints. This study addresses a larger unmet need in neuroscience for new tools to extract insight jointly from behavioral and neural recordings. It also addresses a long running "pixels vs. keypoints" debate in the computational ethology literature for describing behavior with the simple answer to use both.

The PS-VAE uses two modifications of a basic VAE to identify interpretable latent representations of data. First, it splits the ELBO loss function of the VAE into a supervised component that is used to reconstruct labeled keypoints, and an unsupervised component that is used to reconstruct the remainder of variance in the data. Second, additional loss functions are added for the unsupervised space to attempt to 'disentangle' the latent factors. The implementation here appears to be unique, but the development of new disentangling approaches and VAEs with discriminative components is a fast moving space in both machine learning and neuroscience.

The central claim of the manuscript is that the PS-VAE approach detects latent factors with greater interpretability than is possible using existing dimensionality reduction approaches. For instance in a video of a head-fixed mouse, the latent factors appear to describe the movement of defined anatomical regions such as a whisker pad. The challenge in evaluating these claims, as I discuss below, is that they are almost entirely qualitative, and it is not obvious how well this will hold in entirely new domains. While the authors' laudably show examples in three settings, they are all fairly similar across the scope of model organisms and tasks used in neuroscience. Many of the comparisons of the PS-VAE with the VAE are hard to evaluate. In some cases the advantage comes simply from having behavioral labels, and in other cases the PS-VAE serves as ground truth.

The manuscript is well written, scholarly, and fairly polished. I enjoyed the discussion of the literature and found the derivation of the PS-VAE clear and inviting. The code repo is public and integrated with the NeuroCaSS platform and should be a useful asset to the community.

So taken together, I am left feeling like this is a valuable contribution to the literature, but it is not obvious to me how generalizable the results will be, and whether the method will find widespread penetrance. I feel like the most interpretable approach would be to track as many points as possible, and that the applications the PS-VAE will find may be somewhat niche.

<u>Major Points:</u>

(1) Interpretability, especially of the unsupervised factors is poorly defined. It is typically meant here that these factors serve as atomic body parts that are unlabeled (ie Jaw, Whiskers, etc.). But of course one could have interpretable factors that are non-atomic, ie synergistic movements/motifs of the fingers or limbs or entire behaviors.

(2) Relatedly, the fact that PS-VAE unsupervised latents are more interpretable than a VAE is often qualitative, and it is not clear which aspect of the PS-VAE is contributing to the enhancement (disentangling or use of keypoint information). In most cases it seems superior, but it would be natural to compare to a broader set of models to disambiguate whether the interpretability comes from the disentangling in the unsupervised space or the added hand labels. A natural set of comparisons would be to use:

(1) The PS-VAE.
(2) The PS-VAE without unsupervised disentangling terms.
(3) A vanilla VAE with the disentangling terms introduced here (e.g. total correlation and index-code mutual information).
(4) The vanilla VAE.
(5) PCA on the keypoint labels.
(6) PCA on the videos.
(7) PCA on keypoints + PCA on videos.

Of course what would help all of these comparisons is some effective means of quantifying the 'interpretability' of the unsupervised components, rather than relying on qualitative arguments.

(3) Still relatedly, referring to the unsupervised latents of the PS-VAE, and not the VAE by body part name is a bit misleading (e.g. Figure 8). I get the premise is that it would be harder to name the VAE components, but this is a hypothesis. A motivated VAE user might try a bit harder to come up with VAE names. Its possible they are interpretable, but just less atomic than the PS-VAE components. A first step is to label the PS-VAE components as e.g. Latent 0 – "Jaw", but perhaps there is a better second step.
(4) The comparison between the VAE and PS-VAE using the AR-HMM results in particular are a bit tricky to evaluate because the PS-VAE is serving as both ground truth and a comparator. I'm not sure exactly what to take away.
(5) What explains the poor decoding of the motion energy in Fig. S6? Is this at odds with the existing literature?
(6) The conditional VAE is discussed a few times in the text and methods, and some supplementary videos are provided, but there are no benchmarks given and a full description is absent from the text.
(7) You mention posterior collapse of the VAE in discussion of hyperparameters. Does this limit application of the approach to freely moving animals, like the fly videos used in Graving et al.?
(8) There are many discussions of related approaches for conditional/discriminative VAEs and other disentangling loss terms in the text. Can you make any overall statement of novelty about the network in relation to past approaches?
(9) Are there types of video data where the PS-VAE may produce less interpretable components? How general is this ability to all types of video and features?
(10) The enhancement of decoding in Figure 8C is not quantified.

Specific Comments

1. Line 433 – Make comparison with PCA, but although this is likely not necessarily demonstrated.
2. Is Figure 9 in main text or supplement? Fig 9B is referenced first.
3. The latent traversal videos are a bit hard to make sense of since they play so fast. Perhaps lower the framerate?
4. Line 304 – supplementary figures are out of order
5. Fig 9 J,K – labels would help easy interpretation
6. Fig. 8C – missing quantification of the VAE vs PS-VAE
7. What are the pages in the references? They seem wrong in some cases.
