# Supplementary material for: Partitioning variability in animal behavioral videos using semi-supervised variational autoencoders
Source: PLoS Comput Biol. 2021 Sep 22;17(9):e1009439. doi: 10.1371/journal.pcbi.1009439 (PMC8489729; doi:10.1371/journal.pcbi.1009439)
Supplement: Attachment — Submitted filename: PLOS_reviewer_response.pdf [file pcbi.1009439.s049.pdf]

We would like to thank all four reviewers for their thoughtful comments. All agreed that the model introduced in the manuscript is a novel synthesis of supervised and unsupervised models that produces a useful latent behavioral representation. The reviewers also proposed additional analyses and controls to further elucidate the strengths and weaknesses of the model. As a result, we have included several new sections in the manuscript (in addition to many other minor improvements):

1) A Related Works supplement, as requested by reviewers 1, 2 and 3
2) Additional model comparisons, as requested by reviewers 1, 2 and 3
3) The analysis of a freely moving animal, as requested by reviewers 1 and 4
4) The analysis of multiple videos from a common experimental setup that contain different backgrounds, lighting, etc., as requested by reviewer 1

We believe these additions have significantly improved the presentation of our method.

Reviewer #1

The authors present an extension of the VAE which makes use of hand-labeled data in order to produce untangled latent representations that may aid in behavioral quantification. The proposed PS-VAE forces a subset of the latent representations to contain label information and encourage the additional latent dimensions to capture features of behavior that are independent from the labeled inputs.

Overall this work is a very interesting take on feature 'untangling' in low-dimensional latent representations of behavior, and is of interest from both a behavior interpretability a well as deep learning perspective. Movies demonstrating these partitions by generating frames along a single one of the unsupervised dimensions indicate that this method can successfully discover behavioral features from movies that would not be possible using output of posture estimation alone.

The idea of using the supervised and unsupervised subspaces is elegant and explained well in the manuscript and the details of training are sufficient for reproduction. Additionally, the authors have generated and submitted code that appears well-documented.

Here I list a few weaknesses or simply aspects of the manuscript that might be presented more clearly:

Introduction:

The idea of disentangling latent dimensions in image data is not new and prior work might deserve more of a mention in the introduction (Zheng, Zhilin, and Li Sun. "Disentangling latent space for vae by label relevant/irrelevant dimensions." Proceedings of the IEEE/CVF Conference on Computer Vision and Pattern Recognition. 2019, for example).
We thank the reviewer for pointing out this reference; we have included it in the new Related Works supplement.

Results:

The authors mention that traditional methods are limited to constrained tasks, but then only go on to explore videos in head-fixed animals. Demonstrating the viability of this method in a less constrained environment that captures more of the body and a higher diversity of behavior - where simple tracking within the image would not suffice to describe pose - would greatly strengthen these claims.
We agree with this statement, and have included a new section where we fit the PS-VAE to a video of a freely moving mouse after it has been cropped and aligned to egocentric coordinates. We track the ears, nose, back, and tail base, and find unsupervised latents that correspond to different body postures. We hope this addition provides insight into how our model generalizes beyond the head-fixed preparation.

2.2
The comparison of the vanilla VAE results to the true x and y coordinates does not seem like a fair one here and in section 2.3.1. Is it possible to compare the emergent behavior detectors that come from the unsupervised latent space with specifically engineered detectors?
We have now included a comparison between the PS-VAE latents (and the ARHMM states derived from them) with some simple yet specific feature detectors for two of the datasets; these comparisons can be found in the main text and Supplementary Figures S1 and S4.

2.3.2
Authors mention ‘meaningful features’ in the context that small pixel changes be of greater interest than large pixel variance, but do not address the sampling that that could capture rare events of interest. Is this considered in any accuracy metric? For example a rare event that is known to be important to animal but only occurs for a single frame?
This is a subtle point that we did not highlight in the original text; our model will only be able to capture features that comprise a small number of pixels if that feature changes enough throughout the video. For example, we can capture the pupil location because, even though it comprises a small number of pixels, saccades are frequent. If the pupil instead stayed at the same location except for a very small number of frames, we would not expect to capture these changes with high precision. The PS-VAE is more suited to finding latents that represent behavioral features with high variance across the video. We have updated the text in the Results to address this.

The head-fixed example of capturing interpretable unsupervised representations is an exciting addition and should be emphasized - what happens when there are fewer or more unsupervised latent dimensions? How did the authors reach the decision to use only two additional dims? Is it trivial to assign meaning to any number of these latents?
The number of unsupervised latents is an important hyperparameter in our model, and the initial manuscript did not address this directly; we have since updated the Results section to discuss the selection of unsupervised dimensionality in general, and added a new section (Results > Application of the PS-VAE to a head-fixed mouse dataset > Qualitative model comparisons) to

specifically investigate this question with the head-fixed mouse dataset. We were able to find three unique unsupervised dimensions, but unsupervised subspaces larger than this began to replicate information. We hope that this combination of discussion and example provides more insight into this question.

When separating x-y limb motion or mechanical equipment motion, does the method fail when using slightly offset imaging angles?
The imaging angle has no effect on the method. The angle will of course determine how the x-y coordinates are interpreted in physical space, but this will not degrade the quality of the latent representation (assuming the offset does not lead to increased occlusions, etc). For example, the x-y coordinates in the two-view dataset represent different directions in physical space (forward/backward - left/right) than in the head-fixed mouse dataset (forward/backward - up/down). Furthermore, in the two-view dataset we don’t even use the x-y coordinates of the two arms of the mechanical levers; because they move synchronously, we use a 1-dimensional label rather than 4 dimensions (x-y coordinates for both levers). In general the convolutional neural networks used for the encoder/decoder allow us to make arbitrary transformations between the labels and the reconstructed frames.

Discussion:
How is ‘salience of features’ addressed, are there examples where the unsupervised space did not produce immediately interpretable features but may contain information that may be useful to quantify?
In the results presented here we have implicitly defined “salience” as visual recognition of the feature. It is of course possible that features that are not immediately interpretable could still contain useful information, but “useful” is likely dependent on the scientific question at hand. We have included this point in the updated Discussion section.

Again, how relevant is this for phenotyping where individuals may have physical discrepancies in addition to behavioral differences?
This is an excellent point that is related to comments by other reviewers; the latent space will represent not only physical discrepancies, but also differences in background, lighting, etc. To address this concern we have developed an extension of the PS-VAE that accounts for differences between animals by partitioning the latent space into three subspaces: (1) the supervised subspace (as before); (2) the unsupervised subspace (as before); and (3) a new “background” subspace that controls for static differences across sessions (animal appearance, lighting, etc.). Please see the new section (Results > Extending the PS-VAE framework to multiple sessions) for more details.

The authors mention freely moving behavior, but how difficult would it be to apply these methods to recordings with many views where the animal takes up only a small portion of the visual field at any time? Would translating information into an animal-centric coordinate system be feasible before training the PS-VAE? How sensitive would this preprocessing have to be?
We again refer the reviewer to the new section (Results > Application of the PS-VAE to a freely moving mouse), which does in fact use the PS-VAE to analyze a freely moving mouse by first

transforming into animal-centric coordinates. We have done this for a single view, and believe the equivalent process for a multi-view setup will perform similarly, based on our experience with the two-view head-fixed mouse dataset.

Methods:
Is the data used for each example only coming from one individual and one camera configuration? If so are there ways to make this a viable approach for datasets which are recorded across days and individuals and might have drift or lighting differences? If not then this can be made more clear.
Again we refer the reviewer to the new section (Results > Extending the PS-VAE framework to multiple sessions) where we develop an extension of the PS-VAE to solve this problem.

Specific comments:
Line 49 - consist instead of consists?
Thank you for pointing out this error; it has been fixed.

Reviewer #2

Whiteway et al introduce Partitioned Subspace VAEs, a modification of the classical VAE architecture that they show can extract interpretable features of animal movement from tracked videos. PS-VAEs learn a latent representation that separates out video signals arising from tracked features (here, positions of anatomically defined keypoints) from other sources of variance, for example movement of the jaw or chest. They then demonstrate how these identified latents can be further analyzed using AR-HMMs to identify behavioral events, and how latents can be related to neural activity. The authors apply the PS-VAE to three example datasets, in each case contrasting their model with a standard VAE to show the gains in interpretability their model achieves. The authors develop several helpful metrics to demonstrate this contrast, such as the variance of PS-VAE latents aligned to behavioral events. Another strength of this paper is its clear organization and writing, including the Methods section, which does a very nice job of walking the reader through the details of the PS-VAE.

Key points to address:

First, several other papers have proposed semi-supervised versions of VAEs, including modifications to enhance the interpretability of learned latent representations. The authors note the contrast between PS-VAEs and these other methods in the paper Discussion, however I believe the paper needs a more detailed discussion of the differences between the PS-VAE and some of these other models, perhaps by the addition of a Related Works section in the Introduction.
We agree that there is a rich enough VAE/disentangling literature to motivate a dedicated Related Works supplement, which we have now added.

Ideally, it would also be great to also see the PS-VAE compared to some of these other VAE variations (like the pi-VAE) in the Results section, to show what the specific design of the PS-VAE has achieved beyond what was already possible via other methods. Are the results shown here really something that wouldn't be possible with other, related methods? Or is this just the first study to try this kind of analysis on videos from behavioral neuroscience? Alternatively, the authors might include an ablation study to show how the different components of the loss function collectively contribute to the performance of the PS-VAE.
To our knowledge the PS-VAE is the first method of its kind to analyze behavioral videos from neuroscientific experiments in a semi-supervised fashion. The new Related Works supplement makes more explicit the provenance of ideas used in our model, and the novelty of our approach. The pi-VAE is conceptually similar, but does not explicitly represent the label information in the latent space - rather, it conditions the latents on the labels - and thus its goal is slightly different from that of the PS-VAE (i.e. partitioning the subspace into supervised and unsupervised components). We agree that an ablation study is a useful addition, and have included a new section (Results > Application of the PS-VAE to a head-fixed mouse dataset > Qualitative model comparisons) that explores (1) a model without the unsupervised disentangling term, but with the label reconstruction; and (2) a model with the unsupervised disentangling term, and without the label reconstruction. We hope this addition has provided more insight into how different components of the loss function affect the resulting latent representation.

An obvious question that arose in reading this paper was how the results depend on the dimensionality of the unsupervised latent space. This is a user-provided parameter, and in the paper with one exception it's always set to two (the one exception being a case where two supervised latents were removed, and the number of unsupervised latents was increased from two to four to compensate.) How should an end-user select the dimensionality of the unsupervised latent space? Is there a way to tell that you've done a good job? If you over- or under-estimate the dimensionality of the unsupervised latents, does interpretability of the results suffer?
We have now addressed this concern; please see the response to Reviewer 1's similar comment.

In the Discussion, the authors describe several possible extensions of the PS-VAE, including its application to freely behaving animals and to neural data. As a more general extension: does this model require the supervised latents to come from tracked positions of keypoints? Or could these inputs take a more general form? Similarly, must the input to the PS-VAE be video data, or could other signals also be used? I understand that the target audience of this paper is neuroscientists using tracking methods on behavior videos, however the PS-VAE seems general enough that it could be relevant to other types of data. While it would be great to see this (or some of the other proposed extensions) addressed with a brief example in the results section, I understand if the authors consider this to be out of scope. Instead, it might be helpful for the authors to include some kind of general summary/figure on the types of data to which the PS-VAE might be applied.

The model does not explicitly require the labels to be tracked positions of keypoints; they could in general be any variable (continuous or discrete) that might be predicted from the model inputs. These inputs could likewise, in general, be any signal for which the user is interested in finding a low-dimensional, partitioned representation. However, despite the generality of the approach, we agree that demonstrating the model on other types of data is out of the scope of this manuscript. Instead we have slightly expanded this topic in the Discussion, and added featurization of spike waveforms as another possible application that we have been considering.

Finally, I had one more specific comment, motivated by the two-view mouse dataset. Here the authors state that “the PS-VAE provides a substantial advantage over the VAE for any experimental setup that involves moving mechanical equipment.” While I agree that the PS-VAE seems likely to outperform the VAE in most settings, it seems that in order for the PS-VAE to successfully isolate equipment-derived signals, it must be possible to predict the appearance of equipment from one or more tracked keypoints. I would guess that there are some types of equipment (deformable objects like mouse bedding? the black/white patterned balls used for fly-on-a-ball experiments?) where keypoint-based prediction of equipment appearance could fail. I suggest the authors add a section to the Discussion on what kinds of signal can vs cannot be learned by the supervised latents.
Thank you for this perspective; we agree and have added this caveat to the Discussion.

Reviewer #3

The world is hungry for new behavioral measurement and insight extraction techniques. To address this, the authors introduce a new dimensionality reduction tool, the PS-VAE (Partitioned Subspace Variational Autoencoder) that offers the ability to produce interpretable latent factors describing behavioral video data that additionally possesses some hand labeled keypoints. This study addresses a larger unmet need in neuroscience for new tools to extract insight jointly from behavioral and neural recordings. It also addresses a long running “pixels vs. keypoints” debate in the computational ethology literature for describing behavior with the simple answer to use both.

The PS-VAE uses two modifications of a basic VAE to identify interpretable latent representations of data. First, it splits the ELBO loss function of the VAE into a supervised component that is used to reconstruct labeled keypoints, and an unsupervised component that is used to reconstruct the remainder of variance in the data. Second, additional loss functions are added for the unsupervised space to attempt to ‘disentangle’ the latent factors. The implementation here appears to be unique, but the development of new disentangling approaches and VAEs with discriminative components is a fast moving space in both machine learning and neuroscience.

The central claim of the manuscript is that the PS-VAE approach detects latent factors with greater interpretability than is possible using existing dimensionality reduction approaches. For instance in a video of a head-fixed mouse, the latent factors appear to describe the movement

of defined anatomical regions such as a whisker pad. The challenge in evaluating these claims, as I discuss below, is that they are almost entirely qualitative, and it is not obvious how well this will hold in entirely new domains. While the authors' laudably show examples in three settings, they are all fairly similar across the scope of model organisms and tasks used in neuroscience. Many of the comparisons of the PS-VAE with the VAE are hard to evaluate. In some cases the advantage comes simply from having behavioral labels, and in other cases the PS-VAE serves as ground truth.

The manuscript is well written, scholarly, and fairly polished. I enjoyed the discussion of the literature and found the derivation of the PS-VAE clear and inviting. The code repo is public and integrated with the NeuroCaSS platform and should be a useful asset to the community.

So taken together, I am left feeling like this is a valuable contribution to the literature, but it is not obvious to me how generalizable the results will be, and whether the method will find widespread penetrance. I feel like the most interpretable approach would be to track as many points as possible, and that the applications the PS-VAE will find may be somewhat niche.
We agree that tracking many points is generally a good approach (though it comes with significant experimenter cost that we would like to minimize if possible). However, as we point out in the manuscript, there are situations where an exhaustive point-tracking approach is not feasible. For example, tracking algorithms need precise hand labels to achieve robust results, and if a body part of interest is covered by fur or feathers, then precise labeling may not be feasible (e.g. the mouse's chest in Results >The PS-VAE enables targeted downstream analyses > A two-view mouse video).

There are also situations in which a body part is occluded and cannot be directly labeled, even though it contributes to behavioral variability. In the new section (Results > Application of the PS-VAE to a head-fixed mouse dataset > Qualitative model comparisons), where we increase the number of unsupervised dimensions for the head-fixed dataset, we find an unsupervised latent that seems to correspond to elbow position, even though the elbows are occluded on every single frame. Rather, the latent is picking up on the effect of the occluded elbow position on the angle of the visible arms and paws. An alternative is to track multiple points on each arm/paw, which will result in many new, highly correlated dimensions; the single PS-VAE latent acts as a succinct summary of this information.

Regarding generalizability, we have included a new section (Results > Application of the PS-VAE to a freely moving mouse) in which we analyze a freely moving mouse in an open arena, which we think partially addresses this concern by demonstrating the PS-VAE is not limited to head-fixed animals. Nevertheless, the PS-VAE is not suited to every experimental setup, and we now address this point in the Discussion.

Major Points:

(1) Interpretability, especially of the unsupervised factors is poorly defined. It is typically meant

here that these factors serve as atomic body parts that are unlabeled (ie Jaw, Whiskers, etc.). But of course one could have interpretable factors that are non-atomic, ie synergistic movements/motifs of the fingers or limbs or entire behaviors.
We agree that interpretable factors exist beyond the movements of atomic body parts. We now address this point in the Discussion.

(2) Relatedly, the fact that PS-VAE unsupervised latents are more interpretable than a VAE is often qualitative, and it is not clear which aspect of the PS-VAE is contributing to the enhancement (disentangling or use of keypoint information). In most cases it seems superior, but it would be natural to compare to a broader set of models to disambiguate whether the interpretability comes from the disentangling in the unsupervised space or the added hand labels. A natural set of comparisons would be to use:

(1) The PS-VAE.
(2) The PS-VAE without unsupervised disentangling terms.
(3) A vanilla VAE with the disentangling terms introduced here (e.g. total correlation and index-code mutual information).
(4) The vanilla VAE.
(5) PCA on the keypoint labels.
(6) PCA on the videos.
(7) PCA on keypoints + PCA on videos.

Of course what would help all of these comparisons is some effective means of quantifying the 'interpretability' of the unsupervised components, rather than relying on qualitative arguments.
This is an excellent point that we have now addressed in the updated manuscript in the new section (Results > Application of the PS-VAE to a head-fixed mouse dataset > Qualitative model comparisons); we respond to each model in turn below:

(1) Already completed
(2) We have added a discussion of this model (the PS-VAE with beta=1) as well as highlighted quantitative (label reconstruction) and qualitative (latent traversal) results. We hope this makes clear that interpretability comes from *both* the added hand labels and the unsupervised disentangling. By removing the unsupervised disentangling term, we lose interpretability in that space while maintaining interpretability in the supervised space.
(3) This model is the beta-TC-VAE (Chen et al 2018); we have added a discussion of this model, as well as latent traversal results. The inclusion of this model demonstrates that removing the supervised component of the PS-VAE while retaining the unsupervised disentangling can still result in interpretable latents, albeit ones that don't necessarily align with the tracked points.
(4) Already completed
(5) PCA on the keypoint labels is limited to giving us information about the already-tracked body parts; as our goal is to extract information in addition to this tracking output, we omit this model from our comparisons.
(6) This is similar to the vanilla VAE, with less representational power as it is a linear model. We have not explicitly fit this model, but we compare it to the PS-VAE in the Discussion.

(7) The PCA representation of a video will likely contain information about the keypoints (depending on the pixel variance driven by those keypoints and the number of retained PCs); our goal with the PS-VAE is to find an unsupervised representation (akin to nonlinear PCA) that is constructed such that it does not contain keypoint information.

We hope that the new addition of models (2) and (3) make it clear that both the added labels and the unsupervised disentangling contribute to the interpretability of the resulting latents in different ways.

Regarding the quantification of interpretability, this is a difficult question that has been the subject of many papers in the disentangling literature. We chose a disentangling metric that seemed to perform well in the literature, and was applicable to our model - the Mutual Information Gap (MIG; introduced in the beta-TC-VAE paper by Chen et al, 2018). However, after much effort and many variations, we were unable to produce MIG scores that were stable or that matched our qualitative observations, and hence did not use this metric in our final version of the manuscript. The discrepancy in what has been reported in the literature and our own experience could be due to many factors, including the relative homogeneity of our datasets and the size of our latent spaces (models in the literature often have much larger latent subspaces, e.g. >100 dimensions).

(3) Still relatedly, referring to the unsupervised latents of the PS-VAE, and not the VAE by body part name is a bit misleading (e.g. Figure 8). I get the premise is that it would be harder to name the VAE components, but this is a hypothesis. A motivated VAE user might try a bit harder to come up with VAE names. Its possible they are interpretable, but just less atomic than the PS-VAE components. A first step is to label the PS-VAE components as e.g. Latent 0 – “Jaw”, but perhaps there is a better second step.

We believe this is a valid concern, and have replaced the PS-VAE latent names with the pattern [Latent x “name”].

(4) The comparison between the VAE and PS-VAE using the AR-HMM results in particular are a bit tricky to evaluate because the PS-VAE is serving as both ground truth and a comparator. I’m not sure exactly what to take away.

We have now included a comparison between an ARHMM based on the PS-VAE latents, and a second ARHMM based on hand-engineered features meant to capture the behaviors ascribed to the PS-VAE latents. We hope this makes clearer that the PS-VAE-based ARHMMs are indeed capturing the behaviors that we claimed, and allows us to then more easily make comparisons to the VAE-based ARHMMs; these new analyses can be found in Supplementary Figures S1 and S4.

(5) What explains the poor decoding of the motion energy in Fig. S6? Is this at odds with the existing literature?

We are not aware of any work in the existing literature that explicitly decodes the motion energy of these different latents/variables. Musall et al 2019 and Stringer et al 2019 both show that principal components of a face motion energy signal are good predictors of neural activity (encoding rather than decoding), consistent with our finding that neural activity decodes the

whisker pad motion energy well. However, neither of the above studies used the motion energy of pupil diameter or location. We are also decoding these quantities from the primary visual cortex; it is likely that other brain regions contain more information about changes in the pupil features, such as Locus Coeruleus and Superior Colliculus.

(6) The conditional VAE is discussed a few times in the text and methods, and some supplementary videos are provided, but there are no benchmarks given and a full description is absent from the text.
Thank you for noticing this; because of the addition of the related models brought up in point (2) above, we have decided to remove the less-relevant conditional VAE and instead compare to the PS-VAE with beta=1 and the beta-TC-VAE.

(7) You mention posterior collapse of the VAE in discussion of hyperparameters. Does this limit application of the approach to freely moving animals, like the fly videos used in Graving et al.?
Posterior collapse can limit the number of unsupervised latents we find, but does not limit the types of data we can use to fit the model. As an example of this, we have now included a freely moving mouse dataset (Results > Application of the PS-VAE to a freely moving mouse).

(8) There are many discussions of related approaches for conditional/discriminative VAEs and other disentangling loss terms in the text. Can you make any overall statement of novelty about the network in relation to past approaches?
We have added a new Related Works supplement that explicitly addresses this question. In short, the novelty of our approach is in combining discriminative VAEs with an unsupervised disentangling loss term.

(9) Are there types of video data where the PS-VAE may produce less interpretable components? How general is this ability to all types of video and features?
It is of course possible that there are certain experimental setups where the PS-VAE will produce less interpretable components. For example, non-static background features that cannot be tracked (such as bedding in a freely moving rodent video) will be represented in the unsupervised latent space, but likely not in an interpretable manner. We have now addressed this limitation of the approach in the Discussion section.

(10) The enhancement of decoding in Figure 8C is not quantified.
Thank you for pointing out this oversight; we have included a new supplemental figure (Supp Fig. S7) with this quantification.

Specific Comments
1. Line 433 – Make comparison with PCA, but although this is likely not necessarily demonstrated.
We have not made the explicit comparison to PCA, but here we are merely pointing out that the structure of the PS-VAE is fundamentally different than that of PCA; to make this point clearer we have reworded the sentence in question: “In contrast to PCA, *which does not take into account external covariates*, the PS-VAE extracts interpretable pose information through the use

of a discriminative network, as well as automatically discovers additional sources of variation in the video."

2. Is Figure 9 in main text or supplement? Fig 9B is referenced first.
This figure is in the main text (albeit in the Methods); we believe it makes sense to have this figure next to the discussion of how to choose hyperparameters. We have updated the text to reference Fig. 9A first.

3. The latent traversal videos are a bit hard to make sense of since they play so fast. Perhaps lower the framerate?
We have found that the easiest way to absorb the information in these videos is to watch them at a relatively normal frame rate, but constantly looping. We have now mentioned this in the overview page of the supplemental videos.

4. Line 304 – supplementary figures are out of order
We have ordered the supplementary figures by topic (ARHMMs, decoding, hyperparam searches, etc). This makes browsing the supplementary results much easier at the expense of correct ordering in the main text. We would prefer to keep these figures ordered topically, but can rearrange if there is strong opposition to this approach.

5. Fig 9 J,K – labels would help easy interpretation
Thank you for pointing this out, we have updated this figure (as well as the corresponding supplemental figures) with labels.

6. Fig. 8C – missing quantification of the VAE vs PS-VAE
We have included a new supplemental figure (Supp Fig. S7) with this quantification.

7. What are the pages in the references? They seem wrong in some cases.
The pages are automatically generated hyperlinks to where the citation appears in our text; they do not reference the pages within the citations themselves.

Reviewer #4

Dear Editors,

The manuscript under review, entitled "Partitioning variability in animal behavioral videos using semi-supervised variational autoencoders" by Whitway et al, provides a new computational method termed Partitioned Subspace Variational Autoencoder (PS-VAE). PS-VAE is a variation of semi-supervised modelling based on a fully unsupervised variational autoencoder, which provides the benefit of both a supervised and unsupervised component - allowing for a more full description of both supervised pose-estimation and unsupervised analysis of variability that is not captured by pose-estimation.

This is a strong approach for the analysis of pose-estimation based behavioral analysis, which has certainly gained a great deal of popularity with the release of pose-estimation architectures like SLEAP and DLC. However, the applicability of this method to behavior other than highly-controlled head-fixed rodent setups remain to be seen.

However, within head-fixed setups, the authors do a great job of validating their approach. The authors use their method in 3 datasets, including one from the IBL, to address the applicability of their approach across different head-fixed behavioral setups. Their approach, although perhaps only applicable pragmatically to head-fixed mice, will certainly be of interest to labs using head-fixed behavioral preparations.

I am impressed with the approach the authors present in this paper. The overall approach is clear, the documentation is acceptable, and inclusion of examples of varying pose-estimation architectures is welcomed.

I have only one major comments:

Based on the current title, I was expecting a more generalizable approach. I strongly suggest the title be changed to better reflect what is shown in the paper. “Partitioning variability in head-fixed rodent behavioral videos using semi-supervised variational autoencoders” would be a much better title, until the inclusion of data suggesting that this method can be applied to freely moving mouse behaviors.
We agree with this comment, and have addressed it by including a new section (Results > Application of the PS-VAE to a freely moving mouse) where we analyze a freely moving mouse dataset with our approach.
